# Synthesis of Samarium-Based Metal Organic Compound Nanoparticles with Polychromatic-Photoluminescence for Bio-Tissue Fluorescence Imaging

**DOI:** 10.3390/molecules24203657

**Published:** 2019-10-10

**Authors:** Ye Wu, Jiquan Yang, Yingcheng Lin, Jian Xu

**Affiliations:** 1 School of Electrical and Automation Engineering, Jiangsu Key Laboratory of 3D Printing Equipment and Manufacturing, Nanjing Normal University, Nanjing 210046, China; wooye@163.com; 2 Key Laboratory of Dependable Service Computing in Cyber Physical Society of Ministry of Education Chongqing University, College of Microelectronics and Communication Engineering, Chongqing University, Chongqing 400044, China; 3 Division of Electrical and Computer Engineering, Louisiana State University, Baton Rouge, LA 70803, USA

**Keywords:** near-infrared fluorescence, bio-tissue, fluorescence imaging, fluorescence probe molecules, UV-Vis-NIR, wide-optical-range imaging

## Abstract

The development of nanomaterials with special optical window is critical for clinical applications and the optoelectronic industry. In this work, eight kinds of samarium-based metal organic compound nanoparticles (Sm–Fe, Sm–Ga, Sm–Mn, Sm–Na, Sm–Nb, Sm–W, Sm–Cu, and Sm–Al) were synthesized through a solution method. They show polychromatic-photoluminescence spectra extended from the UV to near-infrared (NIR) region when excited by 280 nm, 380 nm, 480 nm, 580 nm, and 785 nm light. They emit direct white light with respect to UV excitation. Tunable white-to-green fluorescence can be achieved by variation of excitation light around 300–400 nm. When they are excited by a 785 nm light source, they show intense fluorescence around 800–1100 nm, which is promising for NIR bio-imaging. Their application in multicolor ultra-wide-range bio-tissue fluorescence imaging is demonstrated by UV (359–371 nm), blue (450–490 nm), green (540–552 nm), and NIR light (central wavelength = 785 nm) excitation with pig kidney tissue samples.

## 1. Introduction

As the field of fluorescence imaging contrast agents, including molecule fluorophores, metal-organic frameworks, and coordination polymers matures, its influence is felt further and further out in the optical window. By now, near-infrared (NIR) window ranging from 700 to 1000 nm is of great interest in biomedical imaging due to its unique advantage in achievable penetration depth. Fluorescence imaging in this NIR window has been widely used in clinical practices, including assessment of vascular flow in grafted tissues [1,2], image-guided oncologic surgery [3], and intraoperative fluorescence imaging [4,5]. However, bio-tissues generally contain complex biofluids. Their optical absorption band can be located at any optical band ranging from ultraviolet-visible (UV-Vis) to NIR. Therefore, the development of imaging contrast agents with broad band photoluminescence covering from UV-Vis to NIR is necessary, whose multicolor fluorescence bands can be flexibly selected for practical bio-tissue imaging and clinical/surgical procedures. 

Samarium-based compounds are interesting lanthanide emitters. They typically show orange/red fluorescence [6,7,8,9,10,11,12,13,14,15,16], which is just at the edge of the visible light and the beginning of the NIR light. Reports of Sm–based compounds showing luminescence other than orange/red are fairly rare in the literature [17,18,19]. Then, a question arises. How can luminescence of these Sm–based compounds be tailored to be in the region of UV, blue, yellow, or NIR light?

The spectroscopic chemistry behind the luminescence of the compounds may be related to ligand-to-metal charge transfer [6,7,8,9,10,11,12,13,14,15,16,17,18,19]. In order to tailor the luminescence of the Sm compound, a special design can be made in the ligands, where the electron can transfer between Sm atoms and the ligands. One possibility is to use an ionic liquid as the solvent for the Sm compound, which can expand the normal red photoluminescence into NIR region (around 950 nm). However, the ionic liquid is very expensive compared with ordinary organic solvents such as ethanol, dimethylformamide, dimethyl sulfoxide, and isopropyl alcohol, which hurdles its practical applications. 

It is reported that the presence of a heterofunctional ligand, namely, the introduction of a second metal center (i.e., uranyl) [13,17], opens up another possibility for tailoring the luminescence of Sm compounds. Sm compounds belong to the lanthanide group, whose photoluminescence is generally generated from f-f transitions [13]. Molar absorptivities are low for these compounds, resulting in inefficient direct excitation of the ion. As such, it is common to fabricate an organic ligand providing a donor, which sensitizes Sm^3+^ luminescence emission. 

For instance, a heterometallic carboxyphosphonate, (UO_2_)_2_(PPA)(HPPA)_2_Sm(H_2_O)·2H_2_O was synthesized using this strategy [17]. The UO_2_^2+^ metal ions bind exclusively to the phosphonate moiety, whereas the Sm^3+^ ions are coordinated by both phosphonate and carboxylate functionalities. Photoluminescence studies show very bright visible and NIR Sm–centered emission upon direct excitation of the uranyl moiety. No emission is observed in the region typical of the uranyl cation, indicating that all energy is either transferred to the Sm^3+^ center or lost to non-radiative processes [17].

Based on this strategy, we may speculate that if we can introduce a second metal ion into the Sm compounds, the heterofunctional ligand may provide a donor to be excited. Subsequently, the photoluminescence of Sm can be expanded to the optical region other than orange/red light. 

Using the idea, in this work, we aimed to combine another metal ion with Sm for construction of sulfur–metal–oxygen moiety. We aimed to capitalize on differences in the affinity of Sm^3+^ ions versus the new metal–oxygen ligand for sulfur–metal–oxygen moiety as well as steric restraints imposed by unique coordination geometries of the metal ions for preparation of heterometallic materials. 

We found out that the use of Fe^3+^, Ga^3+^, Mn^2+^, Na^1+^, Nb^5+^, W^5+^, Cu^2+,^ and Al^3+^ ions was successful in introducing a second metal center to the Sm compounds. The ability to select a second metal using the sulfur–metal–oxygen ligand and the promising application of UV-Vis-NIR bio-tissue fluorescence imaging prompted the solution synthesis of Sm–Fe, Sm–Ga, Sm–Mn, Sm–Na, Sm–Nb, Sm–W, Sm–Cu, and Sm–Al compound nanoparticles. 

X-ray photoelectron spectroscopy (XPS), X-ray diffraction spectroscopy (XRD), and transmission electron microscopy (TEM) were used to characterize these synthesized nanoparticles. Furthermore, we present photoluminescence data confirming that they are showing wide-range and polychromatic fluorescence in UV-Vis-NIR range. They present white light emission when excited by UV light. Also, variation of excitation light around 300–400 nm leads to tunable white-to-green fluorescence. When they are excited by 785 nm, they reveal intense NIR fluorescence ranging from 800 nm to 1100 nm. 

Experiments on multicolor wide-range fluorescence imaging were carried by using pig kidney tissues. Due to their UV-Vis-NIR broad fluorescence, the Sm–Fe, Sm–Ga, Sm–Mn, Sm–Na, Sm–Nb, Sm–W, Sm–Cu, and Sm–Al compound nanoparticles are envisioned to play a significant role in the field of biomedical imaging.

## 2. Results

A solution synthesis method is developed to make the samples. The detailed synthesis procedure can be found in Section 4. 

### 2.1. Characterization

**Figure 1:**
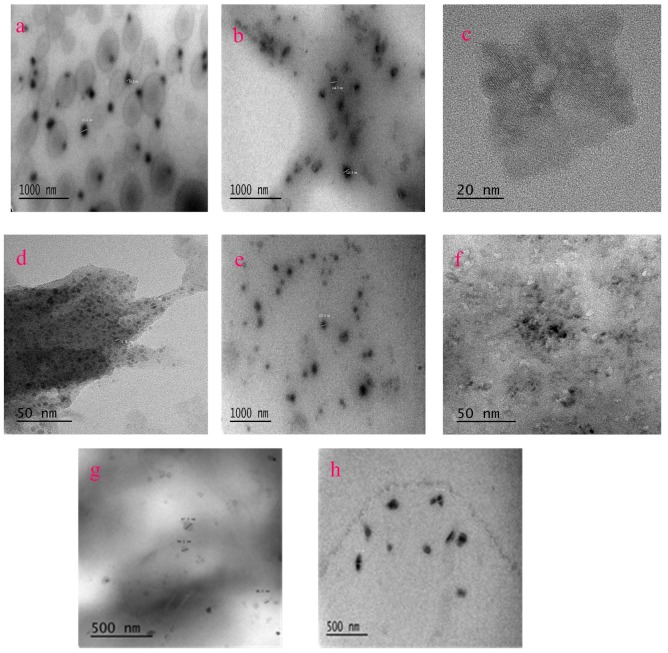
TEM images of Sm–Me (Me = Fe, Ga, Mn, Na, Nb, W, Cu, and Al) metal organic compound nanoparticles: (**a**) Sm–Fe; (**b**) Sm–Ga; (**c**) Sm–Mn; (**d**) Sm–Na; (**e**) Sm–Nb; (**f**) Sm–W; (**g**) Sm–Cu; (**h**) Sm–Al.

TEM images were obtained to examine the scale and morphology of the nanoparticles (Figure 1a–h). All the images show nanoparticle features. Table S1 in the Supplementary Materials lists the size of these nanoparticles, which is obtained after counting 10 pages of TEM images. XRD was used to derive the crystal information of the samples. The results of XRD are listed in the Supplementary Materials (see Figures S1–S2 for crystal cell structures, Figures S3–S10 for simulated and experimental XRD profiles and Table S2 for crystal structure information).

The XPS was applied to study chemical bonding information. The XPS spectra for Sm–Fe were discussed in this section. For lengthy consideration, the XPS spectra for Sm–Ga, Sm–Mn, Sm–Na, Sm–Nb, Sm–W, Sm–Cu, and Sm–Al are presented in the Supplementary Materials (see Figures S11–S17).

**Figure 2:**
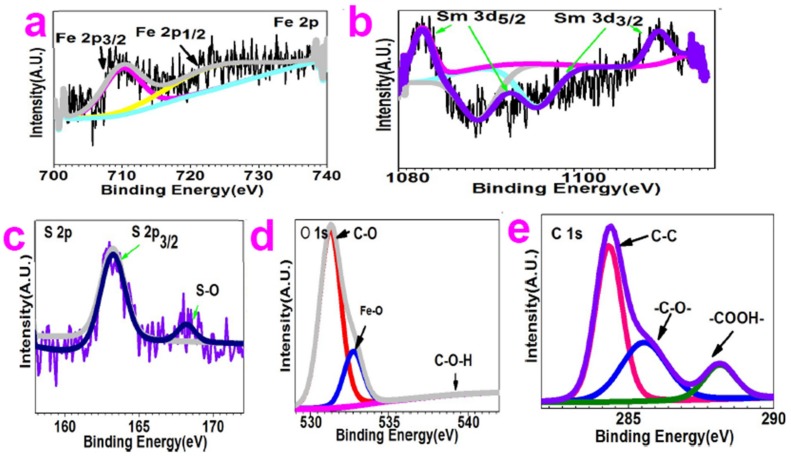
X -ray photoelectron high-resolution scanning spectra of Sm–Fe compound: (**a**) Fe 2p; (**b**) Sm 3d; (**c**) S 2p; (**d**) O 1s; (**e**) C 1s.

Figure 2a depicts the Fe 2p XPS spectra, which show two peaks at 710.1 eV and 724.7 eV, which correspond to the core level of Fe 2p_3/2_ and Fe 2p_1/2_, respectively [20].

Figure 2 plots the Sm 3d XPS spectra, which present six peaks at 1082.7 eV, 1090.6 eV, 1092.6 eV, 1095.6 eV, 1100.8 eV, and 1109.7 eV. The peaks at 1082.7 eV, 1090.6 eV, and 1092.6 eV are all assigned to Sm 3d_5/2_. The peak at 1082.7 eV is the main peak of Sm 3d_5/2_. The peaks at 1090.6 eV and 1092.6 eV correspond to the spin-orbit splitting of the energy levels for Sm 3d_5/2_. The peak at 1109.7 eV is the main peak of Sm 3d_3/2_. The peaks at 1095.6 eV and 1100.8 eV are attributed to spin-orbit splitting of the energy levels for Sm 3d_3/2_ [21,22]. The S 2p XPS spectra (Figure 2c) present two peaks at 163.2 eV and 168.2 eV, which are assigned to S 2p_3/2_ core level and S–O bonding, respectively [23,24].

O 1s XPS spectra (Figure 2d) show peaks at 531.2 eV, 532.7 eV, and 540.1 eV. The peak at 531.2 eV is assigned to C–O bonding [21]. The peak at 532.7 eV is attributed to C–O–H bonding [25,26,27,28]. The peak at 540.1 eV is considered to be due to Fe–O bonding [20,21,27,28].

C 1s XPS spectra (Figure 2e) present peaks at 284.3 eV, 285.6 eV, and 288.2 eV, which are assigned to C–C bonding, C–O bonding, and COOH, respectively [25,26,27,28].

### 2.2. White Light Emission with Tunable White-to-Green Fluorescence by Variation of Excitation Light

**Figure 3:**
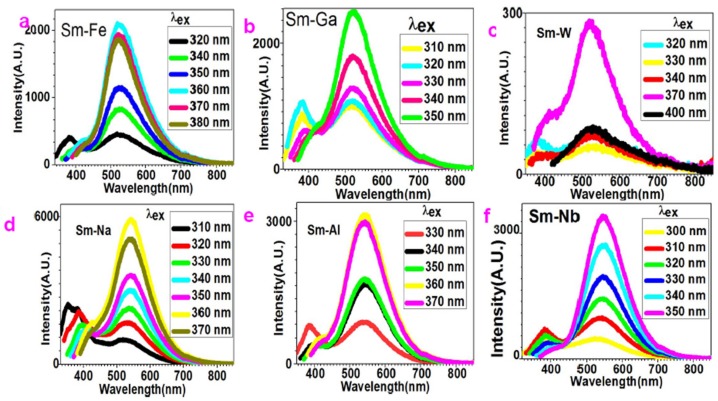
Sm–Fe, Sm–Ga, and Sm–W compound nanoparticles all show tunable white-to-green fluorescence by variation of excitation light around 300–400 nm: (**a**) Sm–Fe; (**b**) Sm–Ga; (**c**) Sm–W; (**d**) Sm–Na; (**e**) Sm–Al; (**f**) Sm–Nb.

As shown in Figure 3a, when excited by 320 nm light, Sm–Fe nanoparticles show white-light emission and two maximum peaks appear at 381 nm and 517 nm. The intensity of these two peaks is comparable. As the wavelength of excitation light increases, the 381 nm peak disappears and the intensity of the 517 nm peak enhances. The fluorescence transits from white light to green light.

Figure 3b depicts the fluorescence spectra of Sm–Ga nanoparticles with the variation of excitation. When excited by 310 nm light, two maximum peaks present at 384 nm and 513 nm. When excited by 320 nm light, the intensity of these two peaks increases at the same time. As the wavelength of the excitation light increases, the intensity of the 384 nm peak is suppressed but that of the 513 nm peak is enhanced. When adjusting the excitation light to 350 nm, the 384 nm peak disappears and only the 513 nm peak is left. This generates green fluorescence finally. It is interestingly to find out that it shows white-to-green transition at the 330 nm light excitation. 

Figure 3c reveals the white-to-green emission of Sm–W nanoparticles. When excited by 320 nm light, two maximum peaks with comparable intensity show at 375 nm and 526 nm. When exited by 330 nm light, both the peaks are suppressed. When excitation wavelength is changed to 340 nm, the peak at 375 nm is suppressed, but the 526 nm peak is enhanced. When excitation light is set to 370 nm, the 375 nm peak almost vanishes. Only a shoulder is left around 375–433 nm and the peak at 526 nm is enhanced. If the excitation light is set to 400 nm, the shoulder completely disappears and only the 526 nm peak is left. At the same time, the intensity of the 526 nm peak dramatically decreases. It shows white-to-green transition at 340 nm light excitation. 

Figure 3d presents the fluorescence of Sm–Na nanoparticles. When excited by 310 nm light, it shows three peaks at 351 nm, 382 nm, and 513 nm. The intensity of the 351 nm and 382 nm peaks is comparable. The intensity of the 351 nm peak is 1.5 times higher than that of the 513 nm peak. The increase in the wavelength of the excitation light suppresses the 351 nm and 382 nm peaks and enhances the 513 nm peak. When excited by 320 nm, it shows two peaks at 385 nm and 531 nm, which is white light emission. When excited by 330 nm, it presents two peaks at 408 nm and 541 nm. When excited by 340 nm, it shows two peaks at 410 nm and 541 nm. When excited by 350 nm, it shows two peaks at 423 nm and 543 nm. When excited by 360 nm, it shows a shoulder around 427 nm and a peak at 542 nm. When excited by 370 nm, the shoulder around 427 nm is almost suppressed and the intensity of the peak at 542 nm decreases. The white-to-green emission transition happens upon 340 nm light excitation.

Figure 3e depicts the white-to-green spectra of Sm–Al nanoparticles. When excitation light of 330 nm is used, two peaks appear at 385 nm and 544 nm with comparable intensity. When excitation light of 340 nm is applied, the peak at 385 nm is suppressed and the peak at 544 nm is enhanced. When the 370 nm excitation light is used, the 544 nm peak stands out from the whole spectrum. The white-to-green emission transition happens when using the 340 nm excitation light.

Figure 3f plots the white-to-green emission of Sm–Nb nanoparticles. When excitation light of 300 nm is employed, two peaks emerge at 384 nm and 532 nm. When the wavelength of the excitation light is up to 310 nm, both the peaks are enhanced. When it is up to 320 nm, the 384 nm peak is suppressed, but the 532 nm peak is enhanced. The enhancement of the excitation wavelength tends to suppress the 384 nm peak and enhance the 532 nm peak. When excitation wavelength is 350 nm, only the 532 nm peak is left. The white-to-green emission transition happens when using the 320 nm excitation light. 

**Figure 4:**
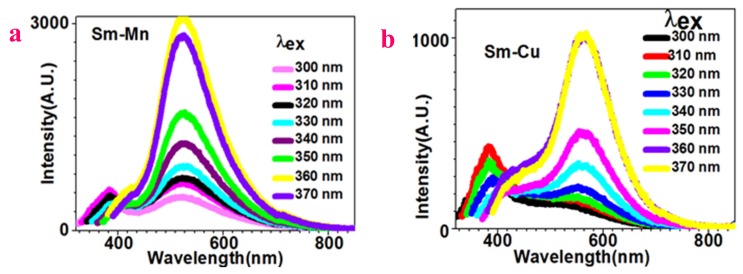
Sm–Mn and Sm–Cu compound nanoparticles show tunable white-to-green fluorescence by variation of excitation light around 300–400 nm: (**a**) Sm–Mn; (**b**) Sm–Cu.

Figure 4a shows the white-to-green emission of Sm–Mn nanoparticles. When excitation light of 300 nm is used, two peaks become apparent at 382 nm and 523 nm. When excitation light of 310 nm is employed, both the peaks are enhanced. When excitation light of 320 nm is applied, the 382 nm peak drops and the 523 nm peak rises. When the wavelength of the excitation light continues to increase, the 382 nm peak is suppressed while the 523 nm peak stands out. The white-to-green emission transition happens when using the excitation light of 330 nm. 

Figure 4b presents the white-to-green emission of Sm–Cu nanoparticles. When the excitation wavelength is set to 300 nm, one peak shows at 384 nm. When it increases to 330 nm, another peak appears at 557 nm. A further increase in the excitation wavelength suppresses the 384 nm peak, but enhances the 557 nm peak. The white-to-green emission transition becomes apparent when using excitation light of 350 nm. 

Indeed, the general procedures of making white-light light-emitting diodes (LEDs) in industry involve using a UV LED light to excite many phosphors or mixing blue LED with a yellow phosphor. It can also be done by packing many LEDs to create a white-light visual sense. However, this brings some issues such as unbalanced color, clumpy layout, and high cost [28]. In order to avoid these issues associated with using multiphosphors or multi-LEDS, the development of single white-light phosphors that can be excited by a single diode is important. It processes a strategic significance in LED industry. Here, it is interesting to find out that the nanoparticles we made emit white light with UV-light excitation. This indicates that they can be used as single white phosphors for making white LEDs equipped with deep UV LEDs, which has a light output at 300–350 nm. 

### 2.3. Polychromatic-Photoluminescence

We varied the wavelength of excitation light as 280 nm, 380 nm, 480 nm, and 580 nm and investigated the fluorescence of these compounds.

**Figure 5:**
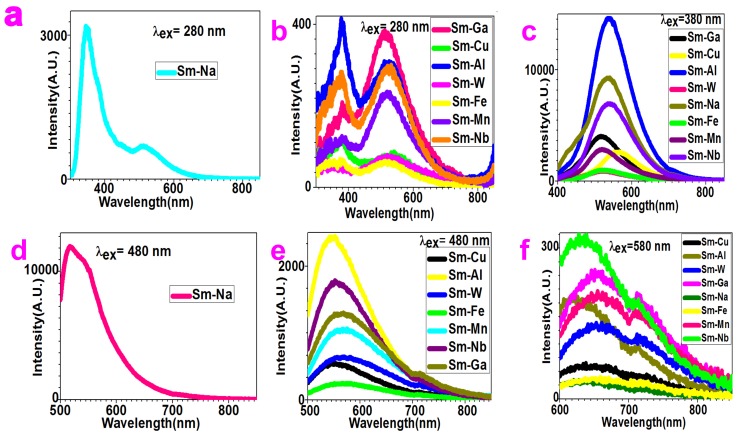
Polychromatic-photoluminescence spectra of Sm–Fe, Sm–Ga, Sm–Mn, Sm–Na, Sm–Nb, Sm–W, Sm–Cu, and Sm–Al compound nanoparticles can be generated by different excitation lights: (**a**)–(**b**) 280 nm; (**c**) 380 nm; (**d**)–(**e**) 480 nm; (**f**) 580 nm.

As shown in Figure 5a, the fluorescence spectra of Sm–Na nanoparticles show two peaks at 344 nm and 511 nm with respect to the 280 nm excitation light. The intensity of the 344 nm peak is three times higher than that of the 511 nm peak. Therefore, Sm–Na nanoparticles present mainly UV emission when excited by 280 nm. 

Figure 5b depicts the fluorescence spectra of Sm–Fe, Sm–Ga, Sm–Mn, Sm–Nb, Sm–W, Sm–Cu, and Sm–Al nanoparticles when the 280 nm excitation light is applied. Here, the fluorescence of Sm–Al shows two peaks at 382 nm and 525 nm. The intensity of the 382 nm peak is higher than the 525 nm peak. The fluorescence of Sm–Nb shows two peaks at 378 nm and 521 nm. The intensity of these two peaks is comparable. The fluorescence of Sm–Ga presents two peaks at 381 nm and 511 nm. The intensity of the 511 nm peak is one time higher than that of the 381 nm peak. The fluorescence of Sm–Mn shows two peaks at 383 nm and 518 nm. The intensity of the 518nm peak is one time higher than that of the 383 nm peak. The fluorescence of Sm–Cu shows two peaks at 381 nm and 540 nm with comparable intensity. The fluorescence of Sm–Fe shows two peaks at 382 nm and 516 nm with comparable intensity. The fluorescence of Sm–W shows two peaks at 379 nm and 519 nm with comparable intensity. All these spectra confirm that Sm–Fe, Sm–Ga, Sm–Mn, Sm–Nb, Sm–W, Sm–Cu, and Sm–Al nanoparticles present white light emission when excited by 280 nm light. 

Figure 5c presents the fluorescence spectra of the Sm compounds excited by 380 nm light. They show peaks at 537 nm (for Sm–Al), 540 nm (for Sm–Nb), 514 nm (for Sm–Ga), 518 nm (for Sm–Mn), 565 nm (for Sm–Cu), 523 nm (for Sm–Fe), 525 nm(for Sm–W), and 535 nm (for Sm–Na). They all present green light emission when excited by 380 nm.

**Figure 6:**
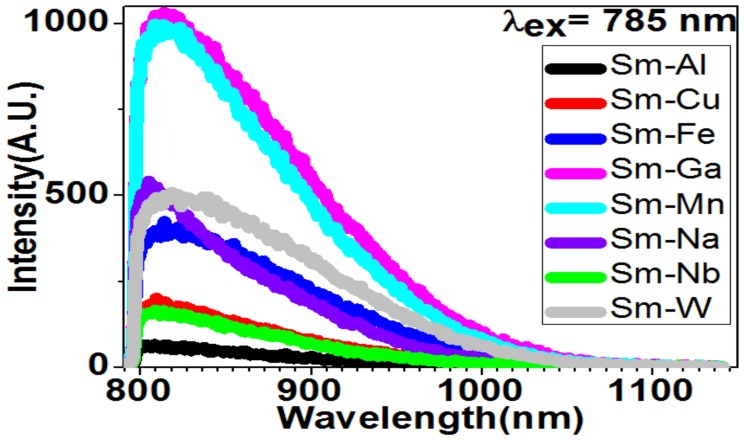
Near-infrared fluorescence for Sm–Me (Me = Fe, Ga, Mn, Na, Nb, W, Cu, and Al) nanoparticles can be generated by 785 nm excitation light.

Figure 5d and Figure 6e compare the fluorescence spectra of the Sm compounds with 480 nm light excitation. They present peaks at 546 nm (for Sm–Al), 552 nm (for Sm–Nb), 565 nm (for Sm–Ga), 569 nm (for Sm–Mn), 547 nm (for Sm–Cu), 568 nm (for Sm–Fe), 564 nm (for Sm–W), and 518 nm (for Sm–Na). It should be noted that the intensity of the 518 nm peak generated by Sm–Na is much higher than that of the other peaks. 

Figure 5f depicts the fluorescence spectra of the Sm compounds with respect to the excitation of 580 nm. They present peaks at 626 nm (for Sm–Al), 634 nm (for Sm–Nb), 657 nm (for Sm–Ga), 665 nm (for Sm–Mn), 651 nm (for Sm–Cu), 671 nm (for Sm–Fe), 660 nm (for Sm–W), 654 nm (for Sm–Na), and a shoulder around 771–824 nm. They all show red light emission when excited by the 580 nm light.

### 2.4. Near-Infrared Fluorescence

NIR fluorescence covering from 800 nm to 1100 nm is observed for the Sm compounds via using 785 nm light excitation (see Figure 6). The NIR fluorescence spectra present peak at 808 nm (for Sm–Al), 812 nm (for Sm–Nb), 813 nm (for Sm–Ga), 815 nm (for Sm–Mn), 809 nm (for Sm–Cu), 814 nm (for Sm–Fe), 817 nm (for Sm–W), and 805 nm (for Sm–Na). It should be noted that the spectrum of Sm–Ga shows the highest intensity. 

**Figure 7:**
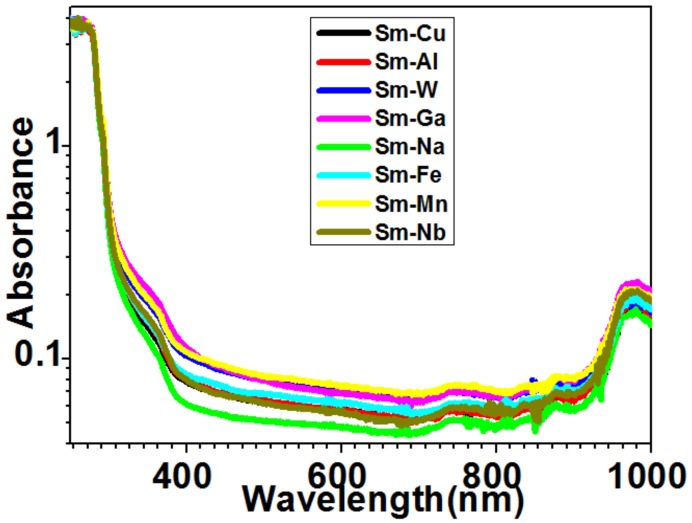
Optical absorption of Sm–Me (Me = Fe, Ga, Mn, Na, Nb, W, Cu, and Al) nanoparticles.

Optical absorption was studied in order to gain more optical information for these compounds. The plot of optical absorption spectra displayed a major shoulder around 250–382 nm, a minor peak around 744 nm, and a major peak around 972 nm (Figure 7). In order to show practical applications of using their UV-Vis-NIR wide-range emission, we carried out fluorescence imaging of pig kidney tissues, which were coated with Sm–Fe, Sm–Ga, Sm–Mn, Sm–Na, Sm–Nb, Sm–W, Sm–Cu, and Sm–Al nanoparticles. 

**Figure 8:**
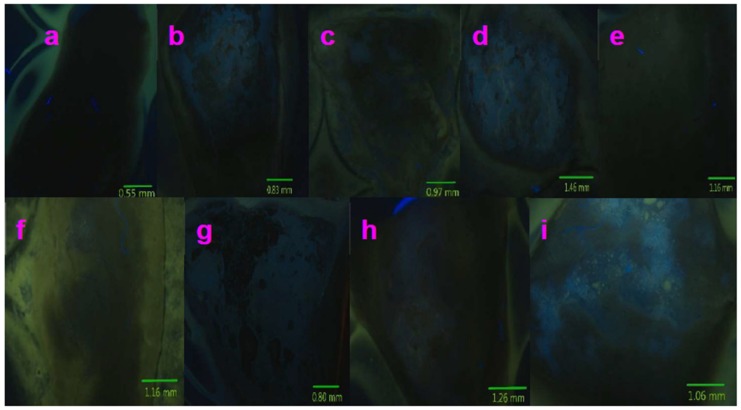
Fluorescence images of pig kidney coated with Sm–Me (Me = Fe, Ga, Mn, Na, Nb, W, Cu, and Al) nanoparticles are generated when UV-light excitation (359–371 nm) is used: (**a**) Control (no nanoparticles are used); (**b**) Sm–Fe; (**c**)Sm–Ga; (**d**) Sm–Mn; (**e**) Sm–Na; (**f**) Sm–Nb; (**g**) Sm–W; (**h**) Sm–Cu; (**i**) Sm–Al.

**Figure 9:**
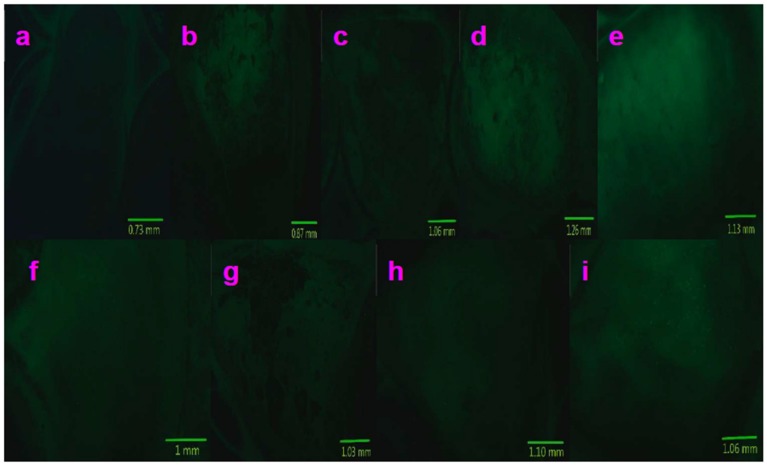
Fluorescence images of pig kidney coated with Sm–Me (Me = Fe, Ga, Mn, Na, Nb, W, Cu, and Al) nanoparticles are generated when blue-light excitation (450–490 nm) is used: (**a**) Control (no nanoparticles are used); (**b**) Sm–Fe; (**c**)Sm–Ga; (**d**) Sm–Mn; (**e**) Sm–Na; (**f**) Sm–Nb; (**g**) Sm–W; (**h**) Sm–Cu; (**i**) Sm–Al.

**Figure 10:**
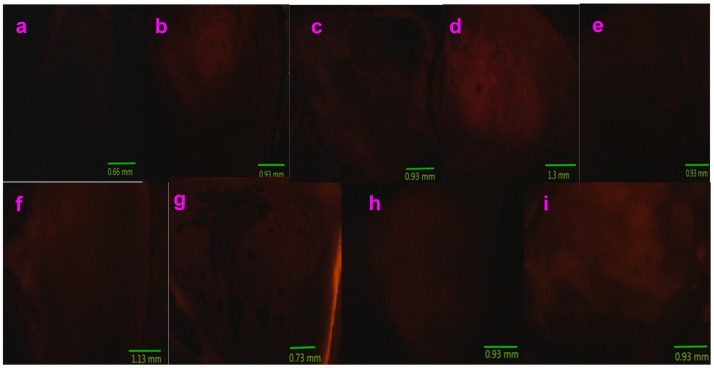
Fluorescence images of pig kidney coated with Sm–Me (Me = Fe, Ga, Mn, Na, Nb, W, Cu, and Al) nanoparticles are generated when blue-light excitation (540–552 nm) is used: (**a**) Control (no nanoparticles are used); (**b**) Sm–Fe; (**c**)Sm–Ga; (**d**) Sm–Mn; (**e**) Sm–Na; (**f**) Sm–Nb; (**g**) Sm–W; (**h**) Sm–Cu; (**i**) Sm–Al.

**Figure 11:**
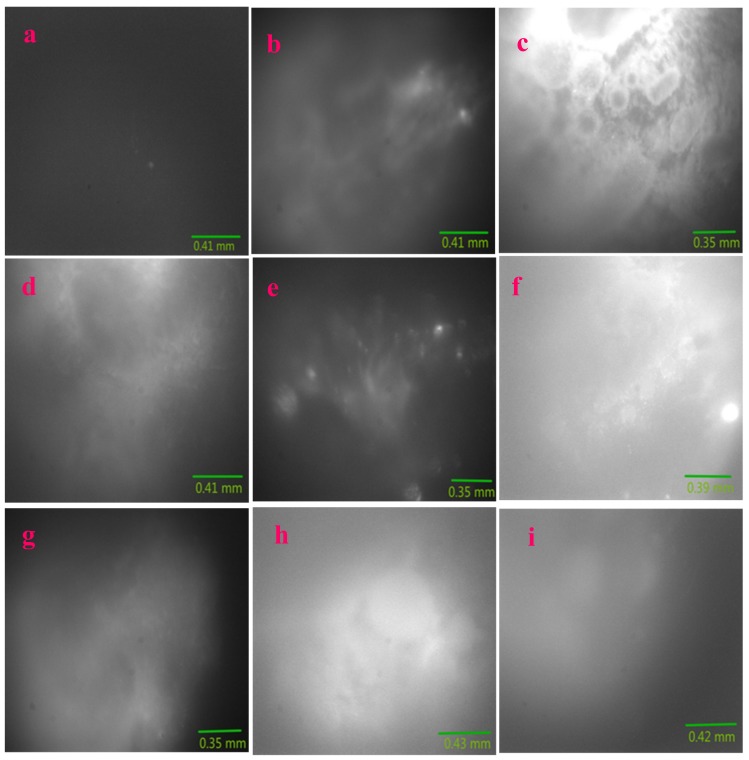
785 nm Laser was used to generate NIR Fluorescence images of pig kidney coated with Sm–Me (Me = Fe, Ga, Mn, Na, Nb, W, Cu, and Al) nanoparticles: (**a**) Control (no nanoparticles are used); (**b**) Sm–Fe; (**c**)Sm–Ga; (**d**) Sm–Mn; (**e**) Sm–Na; (**f**) Sm–Nb; (**g**) Sm–W; (**h**) Sm–Cu; (**i**) Sm–Al.

Figure 8, Figure 9 and Figure 10 present multicolor tissue imaging when UV-light, blue-light, and red-light excitation sources are used. Figure 11 shows the tissue florescence images via using the 785 nm light excitation. These experiments completely examined the impact of the synthesized nanoparticles for the fluorescence imaging of pig kidney in the UV-Vis-NIR optical range.

### 2.5. Fluorescence Imaging of Pig Kidney Tissues

Figure 8a, Figure 9a, and Figure 10a show the control images acquired without using any compounds. It can be observed that very weak fluorescence is generated from the pig kidney itself when excited by UV-, blue-, and red-light, respectively. The spot of the weak fluorescence is due to the existing autofluorescence of the tissue. However, when Sm–Fe, Sm–Ga, Sm–Mn, Sm–Na, Sm–Nb, Sm–W, Sm–Cu, and Sm–Al nanoparticles are used to coat the sample surface, the fluorescence images acquired are much brighter and shaper. They present much more details of the sample surface morphology (Figure 8b–i, Figure 9b–i, and Figure 10b–i). A mountain-valley-like feature can be seen on the tissue surface. 

Figure 11a–i presents NIR fluorescence imaging results of pig kidney tissues upon the 785 nm light excitation. Here, the NIR fluorescence band covers from 800 nm to 1100 nm, which can be seen in Figure 6. The imaging setup is shown in Section 4. 

Figure 11a is the control image of the pig kidney without using any nanoparticles. It turns out that very weak fluorescence in the NIR region is generated in this control image. This is evident in the large region of gray color in Figure 11a. It can be noted that very little feature of the tissue can be found in this cloud-like image. 

Clearly, with the introduction of the Sm–Fe, Sm–Ga, Sm–Mn, Sm–Na, Sm–Nb, Sm–W, Sm–Cu, and Sm–Al nanoparticles into the tissue surface, Figure 11b–i presents very distinctive feature of the sample surface. Microscale features are identified, which are related with the complicated structures of the pig kidney such as blood vessels and tissue surface. 

### 2.6. Optical Chemistry of Sm–Fe, Sm–Ga, Sm–Mn, Sm–Na, Sm–Nb, Sm–W, Sm–Cu, and Sm–Al Compounds

**Figure 12:**
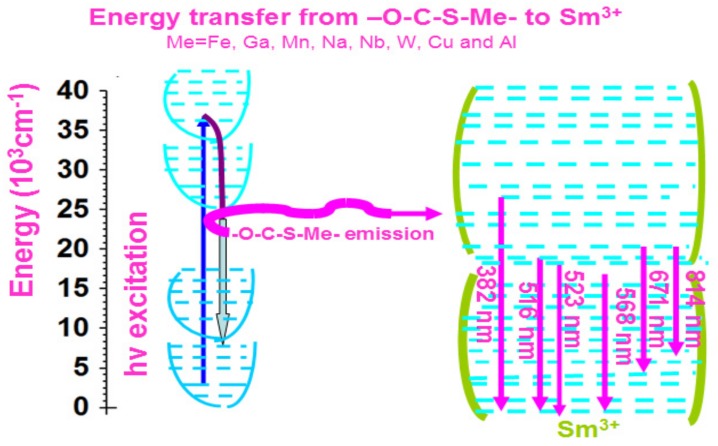
Optical chemistry of Sm–Me (Me = Fe, Ga, Mn, Na, Nb, W, Cu, and Al) compounds for explanation of polychromatic-photoluminescence.

Sm-based compounds are generally reported to show orange/red emission [17,18,19]. Nevertheless, we show eight kinds of Sm compounds i.e., Sm–Fe, Sm–Ga, Sm–Mn, Sm–Na, Sm–Nb, Sm–W, Sm–Cu, and Sm–Al presenting a very wide-range fluorescence covering from UV to NIR. In our synthesized Sm compounds, the Fe^3+^, Ga^3+^, Mn^2+^, Na^1+^, Nb^5+^, W^5+^, Cu^2+,^ and Al^3+^ cations bind to the –S–C–O– moiety with Sm^3+^. It is interesting to note that not only heavy metal ions (e.g., Fe^3+^, Ga^3+^, Mn^2+^, Nb^5+^, W^5+,^ and Cu^2+^) are involved, but also light metal ions such as Na^1+^ and Al^3+^. The –S–C–O– moiety seemingly shows no preferential binding to the heavy metal ions over the light metal ions. We speculate that the –S–C–O– moiety is left completely uncoordinated, thus opening the possibility to introduce a metal center to coordinate with the free –S–C–O– moiety. As shown in Figure 12, a simple optical chemistry diagram is proposed to explain the multiple fluorescence spectra of Sm–Me (Me = Fe, Ga, Mn, Na, Nb, W, Cu, and Al) compounds. We suppose that –S–C–O– combined with Me ions to form the –S–C–O–Me– group in the Sm–Me compounds. It is excited via absorbing excitation photons. The excited –S–C–O–Me– group is used as a sensitizer for the Sm^3+^ activators. This introduces energy transfer from the –S–C–O–Me– group to the Sm^3+^ ion. Then the emission is generated. Various emission lines are observed corresponding with the specific transition of Sm^3+^ ion. 

Here, we take the Sm–Fe compound as an example to discuss the energy level transitions associated with the fluorescence spectra. When it is excited by 280 nm light, the fluorescence spectra of Sm–Fe show two peaks at 382 nm and 516 nm, which are associated with ^6^P→^6^H_5/2_ and ^6^G_7/2_→^6^H_5/2_ transitions of Sm^3+^ ion. When 380 nm light is used as excitation, a peak shows at 523 nm in the fluorescence spectra, which is related with ^4^F_3/2_→^6^H_5/2_ transition; when it is excited by 480 nm light, one fluorescence peak appears at 568 nm, which is attributed to ^4^G_5/2_→^6^H_5/2_ transition. When 580 nm light is applied as excitation, one fluorescence peak presents at 671 nm, which is assigned to ^4^G_7/2_→^6^H_13/2_ transition; when it is excited by 785 nm light, a fluorescence peak appears at 814 nm, which is related with ^4^G_7/2_→^6^F_7/2_ transition [29,30].

The optical chemistry of other compounds is similar. The –S–C–O–Me– group works as a sensitizer for the Sm^3+^ ion activator. The only difference is the emission light and energy level transitions corresponding to the Sm^3+^ ion.

## 3. Discussion

The investigated Sm–Fe, Sm–Ga, Sm–Mn, Sm–Na, Sm–Nb, Sm–W, Sm–Cu, and Sm–Al nanoparticles show a direct white-light emission. This white-light emission transits to green emission via variation of excitation light. If some UV LEDs that have light output from 300–400 nm are applied as excitation light sources, these materials are promising to be used single white phosphors for making white LEDs. The rapid fabrication technology through simple chemistry enables them favorable to be used in the lighting industry.

A laser is generally made by a pumping source, a gain medium, and mirrors with specific optical transmission and reflection. The pumping source and mirrors can normally be purchased from the market. However, the gain medium is not always obtainable. As the key component of the laser, the gain medium decides the output wavelength of the laser. Here, these nanoparticles we made show an impressive wide-range emission covering from UV to NIR when different excitation lights such as 280 nm, 380 nm, 480 nm, 580 nm, and 785 nm are used. This makes them appropriate to be used as raw material systems for making new lasers similar to dye lasers. Novel lasers can be made by combining these materials with an optical parametric amplifier (OPA) that can generate light of variable wavelengths and be used as a pumping source. These lasers are envisioned to show tunable output-wavelengths by using these materials as gain mediums.

Another promising application of these Sm-based nanoparticles would be shown in the field of biomedical imaging. We have shown this potential application for these materials through fluorescence imaging of pig kidney tissues. The development of these imaging contrast agents with wide photoluminescence is useful, given that fluorescence bands can be flexibly selected for practical clinical imaging with different tissues. Our future endeavors would be focused on temperature-depended photoluminescence, quantum yield measurement, and the calculation of π-electron HOMO-LUMO electronic transition, which can provide more information about these compounds in the molecular level.

## 4. Materials and Methods

### 4.1. Synthesis of Sm–Me (Me = Fe, Ga, Mn, Na, Nb, W, Cu, and Al) Nanoparticles

All the raw materials were purchased from Alfa Aesar. The following describes the synthesis in detail:

(a) Preparation of Solution 1: l-cysteine (2 g) was dissolved in deionized water (50 mL).

(b) Preparation of Solution 2: an organic mixture was first prepared by mixing l-cysteine oxalic acid dehydrate (5 g), 2-methylimidazole (2 g), teraphthalic acid (5 g), fumaric acid (5 g), 2,5-dihydroxyterephthalic acid (0.5 g), D(+)-glucose (5 g), dimethyl sulfoxide (20 mL), methacrylic anhydride (10 mL), dimethylformamide (300 mL), oleic acid (40 mL), and diethylene glycol (40 mL). This mixture was stirred to get a clear solution (Solution 2). Solution 2 and Solution 1 were mixed and stirred for 4 h in a closed bottle to get an organic solution (Solution 3).

(c) For preparing the Sm–Fe compound, samarium (III) chloride (0.4 g) was mixed with iron (III) chloride (0.1 g) and stirred in water (5 mL) to get a clear solution containing Sm and Fe ions. This Sm–Fe solution was mixed with Solution 3 (50 mL). Then the mixture was heated at a temperature of 120 °C for 1.5 h in a closed bottle to get a black-red solution. It was centrifuged at a speed of 8000 rpm for 30 min. The sediments left at the bottom of the centrifuge tube were discarded. The solution of the top layer in the centrifuge tube was taken out and transferred to a new centrifuge tube, which was centrifuged at a higher speed of 10,000 rpm for 30 min. The solution of the top layer was taken out and the acquired sediments were dumped again. The acquired solution was transferred to another centrifuge tube and centrifuged at a speed of 14,000 rpm for 30 min to get the final Sm–Fe compound nanoparticles. Here, the purpose of centrifuging at different speeds is to get rid of the residuals in the chemical reaction.

(d) The preparation of Sm–Ga, Sm–Mn, Sm–Na, Sm–Nb, Sm–W, Sm–Cu, and Sm–Al is similar to that of the Sm–Fe compound. The only difference is the usage of gallium (III) chloride (0.1 g), manganese (II) chloride (0.1 g), sodium chloride (0.1 g), niobium (V) chloride (0.1 g), tungsten hexachloride (0.1 g), copper (II) chloride (0.1 g), and aluminum chloride (0.1 g).

### 4.2. Instruments

The XPS data were collected through a K-Alpha XPS instrument (Thermo Scientific, Inc., Waltham, Massachusetts, MA, USA). UV-Vis fluorescence imaging of the samples was carried out via a Zeiss Lumar fluorescence microscope with UV-light (359–371 nm), blue-light (450–490 nm), and green-light (540–552 nm) excitation. Powder XRD characterization data were collected through a Panalytical Empyrean XRD instrument. TEM study was conducted via a JEOL 1400 TEM (120 kV). The UV-Vis-NIR absorption and UV-Vis fluorescence spectroscopy data were collected through a Spark spectrometer.

### 4.3. Experimental Setup of Fluorescence Spectroscopy and Fluorescence Imaging

**Figure 13:**
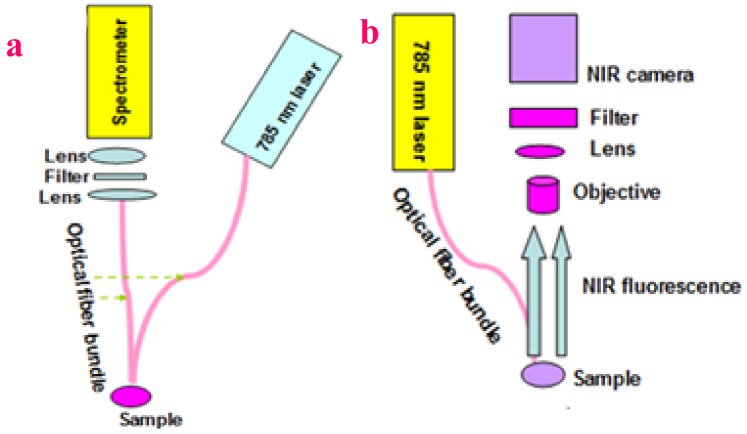
(**a**) Set-up for near-infrared fluorescence spectra measurement. (**b**) Near-infrared fluorescence imaging setup.

NIR fluorescence spectroscopy and fluorescence imaging were performed through a lab-built set-up. A detailed drawing of the setup schematic was shown in Figure 13a,b. The setup is described as follows:

For NIR fluorescence spectroscopy (see Figure 13a), a laser diode (center wavelength = 785 nm, Thermo Scientific, Inc., Waltham, Massachusetts, USA) was applied for providing excitation light. The fluorescence from the sample was collected through an optical fiber bundle. An NIR long-pass filter was positioned in front of an NIR spectrometer (Ocean Optics, Inc., Dunedin, Florida, FL, USA.) to eliminate the excitation light. A lens was used to focus the collected fluorescence into the inlet of the spectrometer.

For NIR fluorescence imaging (see Figure 13b), the same laser diode with center wavelength 785 nm was used for providing excitation light. The sample was mounted on a platform that can be moved in the X-, Y-, and Z-direction. An NIR camera was placed in the direction perpendicular to the sample surface. An NIR long-pass filter was placed in front of the NIR camera to get rid of the excitation light. After the sample surface was irradiated by the laser, the fluorescence image of the sample surface was captured by the camera.

A pig kidney, bought from a local Asian supermarket in Baton Rouge, USA, was used as a sample for the study of UV-Vis-NIR fluorescence tissue imaging. It was cut into many pieces. The dimension of every piece was around 3 × 4 × 2 mm^3^. One drop of the synthesized Sm compound nanoparticle solution was coated onto the surface of the pig kidney pieces. Then, after waiting for around 2–3 min, the pig kidney pieces were used in the fluorescence imaging experiments.

## 5. Conclusions

We synthesized Sm–Fe, Sm–Ga, Sm–Mn, Sm–Na, Sm–Nb, Sm–W, Sm–Cu, and Sm–Al metal organic compound nanoparticles using simple chemistry. The nanoparticles were characterized by XRD, XPS, and TEM. They exhibited polychromatic fluorescence in the optical region of UV-Vis-NIR when excited by 280 nm, 380 nm, 480 nm, 580 nm, and 785 nm light. When excited by 785 nm, they revealed NIR fluorescence ranging around 800–1100 nm. Furthermore, transition with tunable white-to-green fluorescence can be observed by variation of the excitation light around 300–400 nm. This indicates their promising use as single white phosphors for making white LEDs with UV-light LD pumping. Their ability for multicolor bio-tissue fluorescence imaging was demonstrated by using pig kidney samples. In future applications, these novel metal organic compounds can be expected to play a vital role in wide-range bio-tissue fluorescence imaging and used as gain mediums for making tunable lasers.

## Supplementary Materials

The following are available online at https://www.mdpi.com/1420-3049/24/20/3657/s1, Figure S1: Crystal cell structure of Sm–Fe, Sm–Ga, Sm–Na, and Sm–Nb compounds is illustrated by spheres of different color, Figure S2: Crystal cell structures of Sm–W, Sm–Al, and Sm–Mn compounds are illustrated by spheres of different color, Figure S3: Simulated and experimental XRD profiles of Sm–Fe compound, Figure S4: Simulated and experimental XRD profiles of Sm–Ga compound, Figure S5: Simulated and experimental XRD profiles of Sm–Mn compound, Figure S6: Simulated and experimental XRD profiles of Sm–Na compound, Figure S7: Simulated and experimental XRD profiles of Sm–Nb compound, Figure S8: Simulated and experimental XRD profiles of Sm–W compound, Figure S9: Simulated and experimental XRD profiles of Sm–Al compound, Figure S10: Simulated and experimental XRD profiles of Sm–Cu compound, Figure S11: XPS spectra of Sm–Ga compound: (a) Ga 3d; (b) Sm 3d; (c) S 2p; (d) O 1s; (e) C 1s, Figure S12: XPS spectra of Sm–Mn compound: (a) Mn 2p; (b) Sm 3d; (c) S 2p; (d) O 1s; (e) C 1s, Figure S13: XPS spectra of Sm–Na compound: (a) Na 1s; (b) Sm 3d; (c) S 2p; (d) O 1s; (e) C 1s, Figure S14: XPS spectra of Sm–Nb compound: (a) Nb 3d; (b) Sm 3d; (c) S 2p; (d) O 1s; (e) C 1s, Figure S15: XPS spectra of Sm–W compound: (a) W 4f; (b) Sm 3d; (c) S 2p; (d) O 1s; (e) C 1s, Figure S16: XPS spectra of Sm–Cu compound: (a) Cu 2p; (b) Sm 3d; (c) S 2p; (d) O 1s; (e) C 1s, Figure S17: XPS spectra of Sm–Al compound: (a) Al 2p; (b) Sm 3d; (c) S 2p; (d) O 1s; (e) C 1s, Table S1: Table S1: Size of Sm–Fe, Sm–Ga, Sm–Mn, Sm–Na, Sm–Nb, Sm–W, Sm–Cu and Sm–Al nanoparticles, Table S2: Crystal structure information of Sm–compound.
