# Supplementary material for: Synthesis of Samarium-Based Metal Organic Compound Nanoparticles with Polychromatic-Photoluminescence for Bio-Tissue Fluorescence Imaging
Source: Molecules. 2019 Oct 10;24(20):3657. doi: 10.3390/molecules24203657 (PMC6832984; doi:10.3390/molecules24203657)
Supplement: Supplementary file 1 [file molecules-24-03657-s001.pdf]

Supportive information

Synthesis of samarium based metal organic compound nanoparticles with polychromatic-photoluminescence for bio-tissue fluorescence imaging

Ye Wu, Jiquan Yang, Yingcheng Lin and Jian Xu

Section 1. Size of the nanoparticels.

Section 2. Crystal information.

Section 3. XPS analysis.

Section 1. Size of the nanoparticles.

Table S1. Size of Sm-Fe, Sm-Ga, Sm-Mn, Sm-Na, Sm-Nb, Sm-W, Sm-Cu and Sm-Al nanoparticles

Nanoparticles name	Size
Sm-Fe	103 nm
Sm-Ga	70 nm
Sm-Mn	40 nm
Sm-Na	100 nm
Sm-Nb	96 nm
Sm-W	13 nm
Sm-Cu	5 nm
Sm-Al	70 nm

Section 4: Crystal information.

XRD was used to derive the crystal information of the samples. The detailed crystal structure information can be found in Supportive Information (see Table S2). The crystal structure was drawn by using DRAWxtl 5.5 software package (see Figure S1, S2). The simulated and experimental XRD profile is shown in Figure S3-S10.

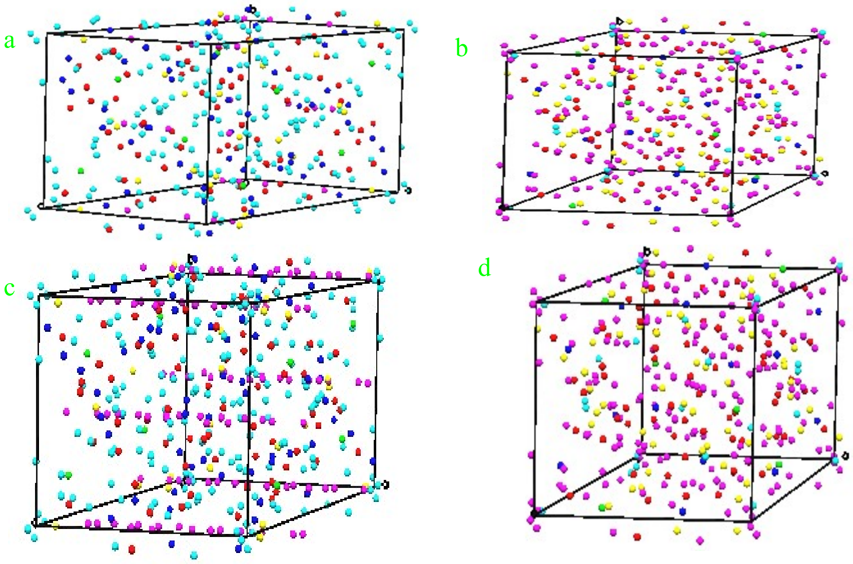

Figure S1. Crystal cell structure of Sm-Fe, Sm-Ga, Sm-Na and Sm-Nb compounds is illustrated by spheres of different color. (a) H: red, Sm: green, C:blue, S: yellow, O:cyan, Fe: Magenta. (b) H: red, Ga: green, Sm: blue, C: yellow, O: Magenta, S: Cyan. (c) H: Red, Sm: green, C:blue, S: yellow, O:cyan, Na:Magenta. (d) H: red, Nb:green, Sm: blue, C: yellow, S: cyan, O: magenta.

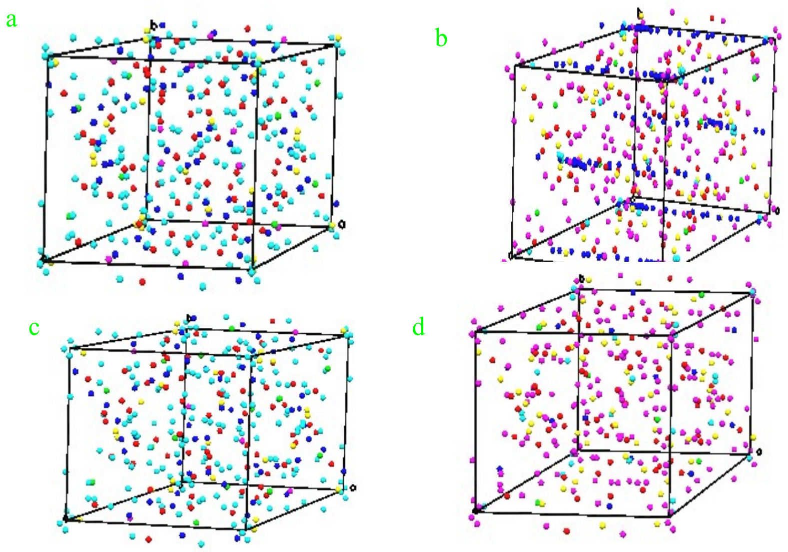

Figure S2 Crystal cell structures of Sm-W, Sm-Al and Sm-Mn compounds are illustrated by spheres of different color. (a) H: red, Sm: green, C: blue, S: yellow, O: cyan, W: Magenta. (b) H: Red, Sm: green , Al : blue, C: yellow, S: cyan, O: magenta. (c) H: red, Sm: green, C: blue, S: yellow, O: cyan, Mn:magenta. (d) H: red, Cu: green, Sm: blue, C: yellow, S: cyan, O: magenta.

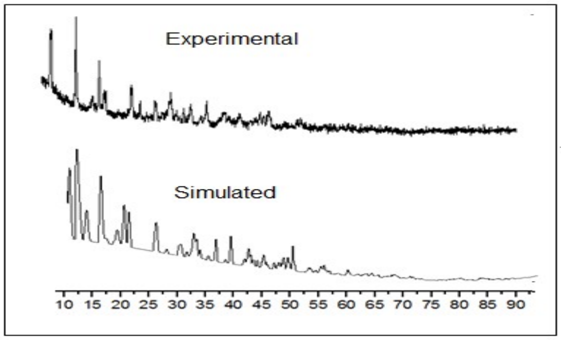

Figure S3. Simulated and experimental XRD profiles of Sm-Fe compound.

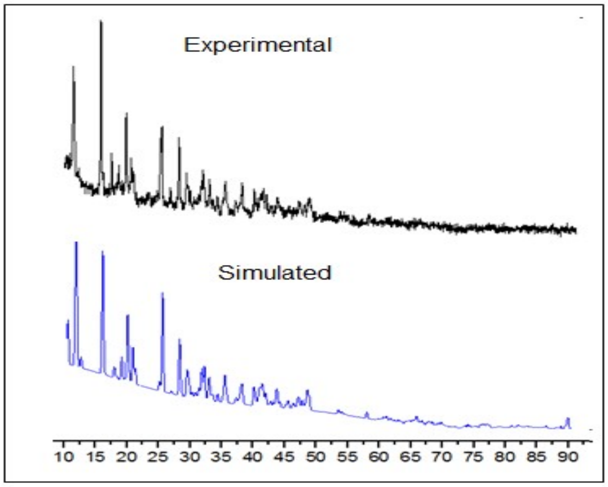

Figure S4. Simulated and experimental XRD profiles of Sm-Ga compound.

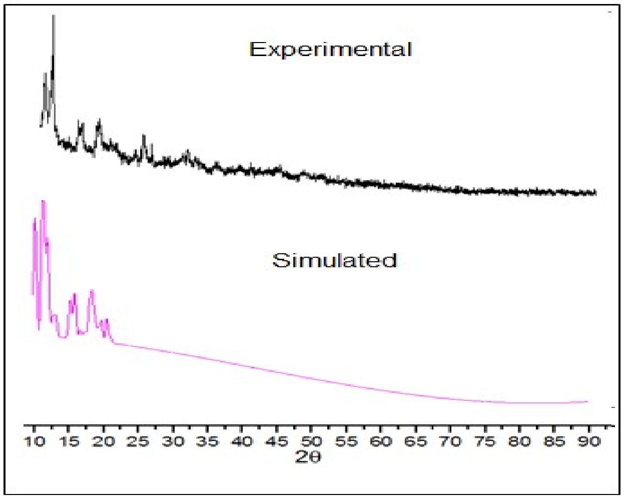

Figure S5. Simulated and experimental XRD profiles of Sm-Mn compound.

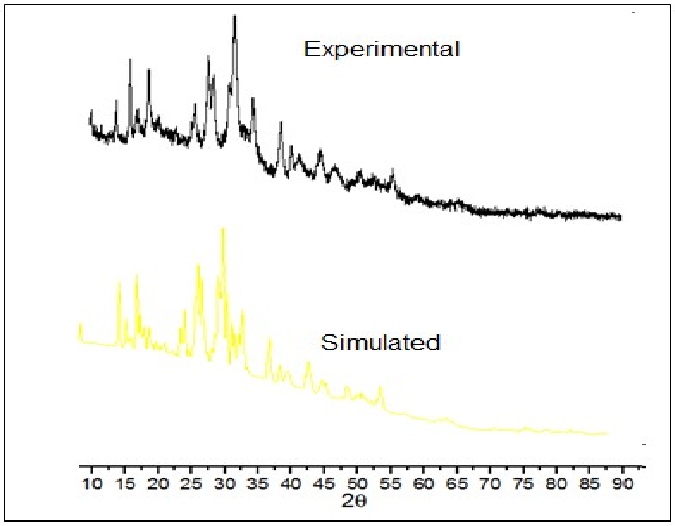

Figure S6. Simulated and experimental XRD profiles of Sm-Na compound.

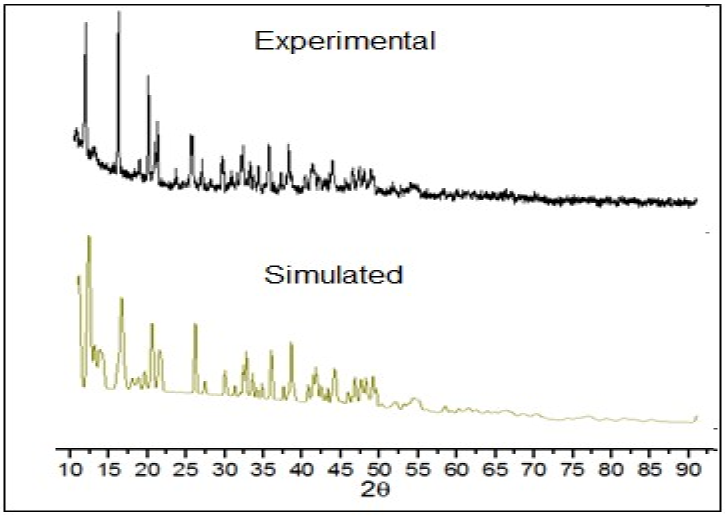

Figure S7. Simulated and experimental XRD profiles of Sm-Nb compound.

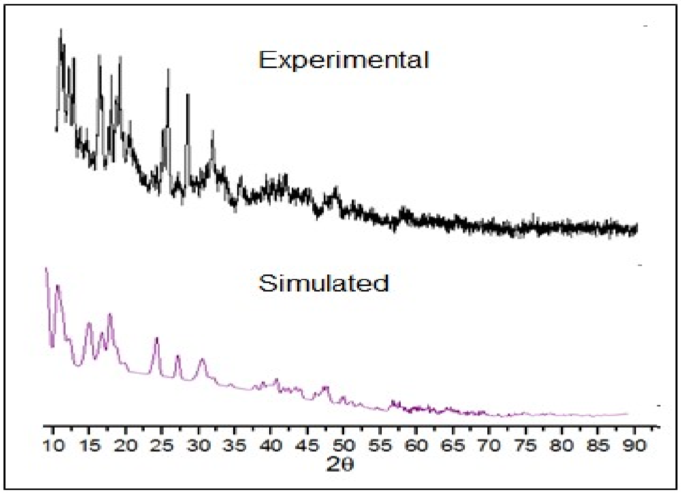

Figure S8. Simulated and experimental XRD profiles of Sm-W compound.

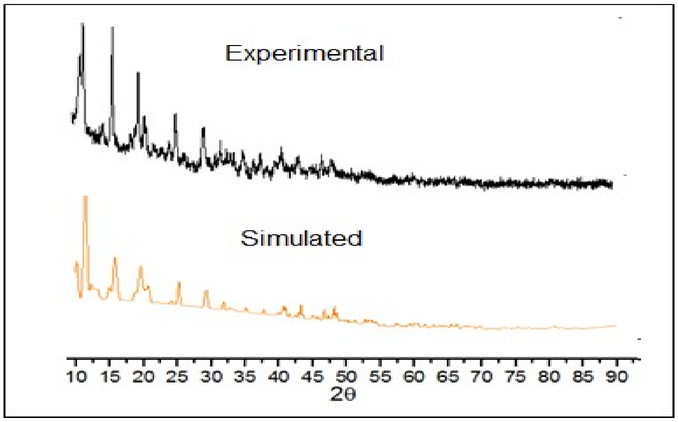

Figure S9. Simulated and experimental XRD profiles of Sm-Al compound.

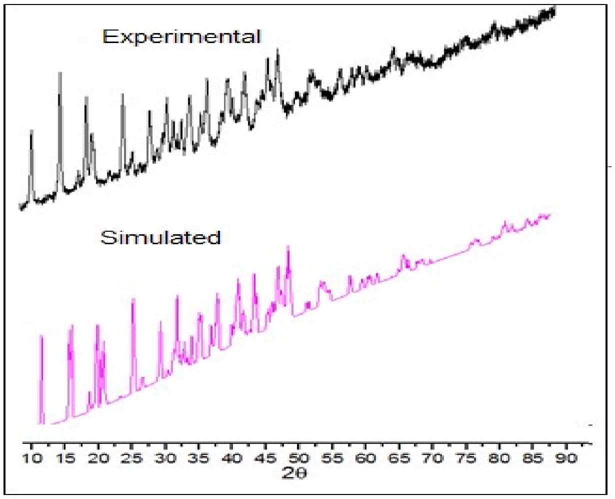

Figure S 10. Simulated and experimental XRD profiles of Sm-Cu compound.

Table S 2: Crystal structure information of Sm-compound.

	Cell length	Cell angle	Formula	Space group	Symmetry
Sm-Fe	a=10.856,b=10.299, c=20.101	α=90.000,β=103.729, γ=90.000	$C_6H_8Fe_4O_{21}S_3Sm$	P 21/c	monoclinic
Sm-Ga	a=11.954,b=10.362, c=20.572	α=90.000,β=103.572, γ=90.000	$C_6H_8GaO_{21}S_3Sm_2$	P 21/c	monoclinic
Sm-Mn	a=11.604,b=10.496, c=20.591	α=90.000,β=102.461, γ=90.000	$C_6H_8MnO_{21}S_3Sm_2$	P 21/c	monoclinic
Sm-Na	a=11.881,b=10.341, c=20.634	α=90.000,β=103.418, γ=90.000	$C_6H_8Na_{15}O_{21}S_3$	P 21/c	monoclinic
Sm-Nb	a=12.095,b=10.495, c=20.857	α=90.000,β=103.293, γ=90.000	$C_6H_8NbO_{21}S_3Sm_2$	P 21/c	monoclinic
Sm-W	a=11.945,b=10.375, c=20.574	α=90.000,β=103.475, γ=90.000	$C_6H_8O_{21}S_3SmW_2$	P 21/c	monoclinic
Sm-Cu	a=12.374,b=10.710, c=21.311	α=90.000,β=103.537, γ=90.000	$C_6H_8CuO_{21}S_3Sm_2$	P 21/c	monoclinic
Sm-Al	a=12.129,b=10.306, c=20.566	α=90.000,β=103.425, γ=90.000	$C_6H_8Al_{25}O_{21}S_4Sm$	P 21/c	monoclinic

Section 5: XPS analysis.

The following section will give a detailed analysis of XPS spectra for Sm-Ga, Sm-Mn, Sm-Na, Sm-Nb, Sm-W, Sm-Cu and Sm-Al compound. (Note: the references for the following section are listed at the end of the Supportive Information). Sm-Fe XPS spectra was shown in the main section of the manuscript.

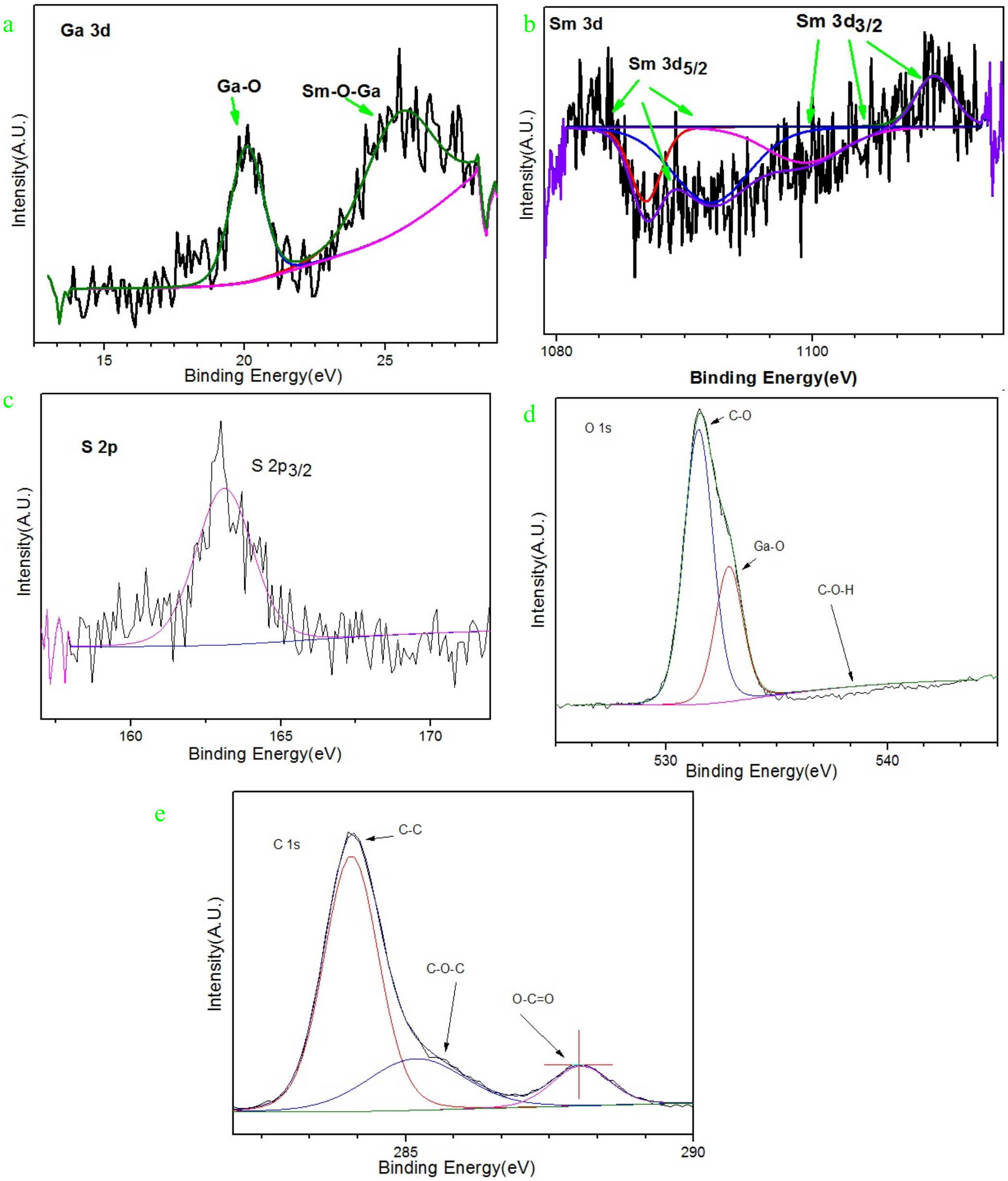

Figure S11. XPS spectra of Sm-Ga compound: (a) Ga 3d; (b) Sm 3d; (c) S 2p; (d) O 1s; (e) C 1s.

Figure S11 shows the XPS spectra for Sm-Ga compound. Ga 3d XPS spectra show two peaks at 20.1 eV, 25.7 eV. The peak at 20.1 eV is assigned to Ga-O bonding.[s13] The peak at 25.7 eV is possibly due to Sm-O-Ga bonding. Sm 3d XPS spectra show peaks at 1083.5 eV, 1089.3 eV, 1091.6 eV, 1096.8 eV, 1099.8 eV, 1109.7 eV. The peaks at 1083.5 eV, 1089.3 eV, 1091.6 eV are due to Sm $3d_{5/2}$. Actually, the peak at 1083.5 eV is corresponding to the core level of Sm $3d_{5/2}$.[s2,s3] The peaks at 1089.3 eV,1091.6 eV are regarded as the spin-orbit splitting of the energy

levels for Sm $3d_{5/2}$.[s2,s3] The peaks at 1096.8 eV, 1099.8 eV,1109.7 eV are related with Sm $3d_{3/2}$. The peak at 1109.7 eV is the main peak and related with the core level of Sm $3d_{3/2}$.[s2,s3] The peaks at 1096.8 eV, 1099.8 eV are related with the spin-orbit splitting of the energy levels for Sm $3d_{3/2}$.[s2,s3] S 2p XPS spectra show peak at 163.1 eV, which is related with S $2p_{3/2}$ core level.[s4,s5] O 1s spectra present peaks at 531.4 eV and 532.8 eV, and a shoulder around 535.2 -543.7eV. The peak at 531.4 eV is attributed to C-O bonding. [s10] The peak at 532.8 eV is due to Ga-O bonding and the shoulder is assigned to C-O-H bonding.[s11,s12] C 1s XPS spectra show peaks at 284.1 eV, 285.2 eV and 288.1 eV, which are assigned to C-C bonding, C-O-C bonding, O-C=O bonding, respectively. [s10]

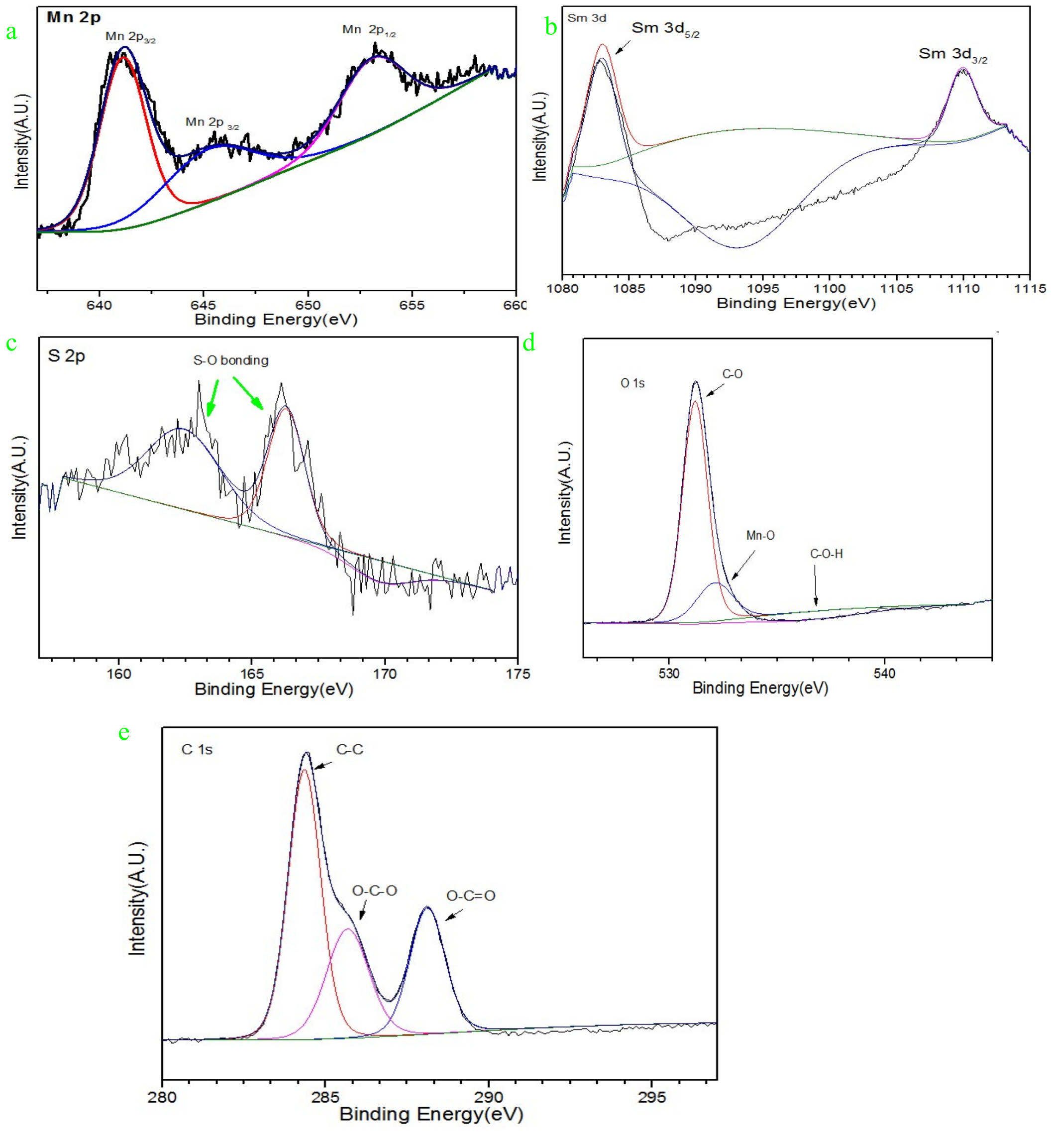

Figure S 12. XPS spectra of Sm-Mn compound: (a) Mn 2p; (b) Sm 3d; (c) S 2p; (d) O 1s; (e) C 1s.

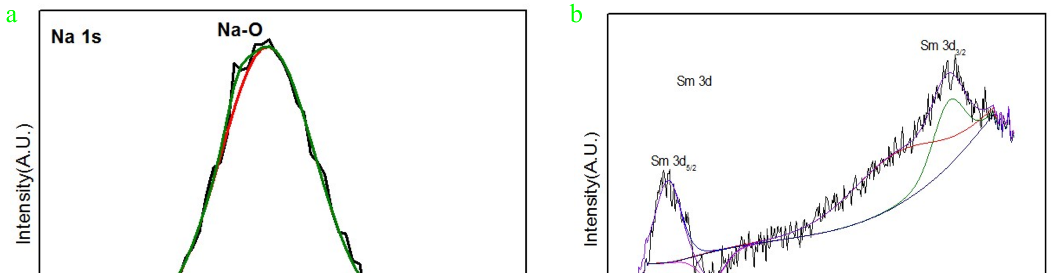

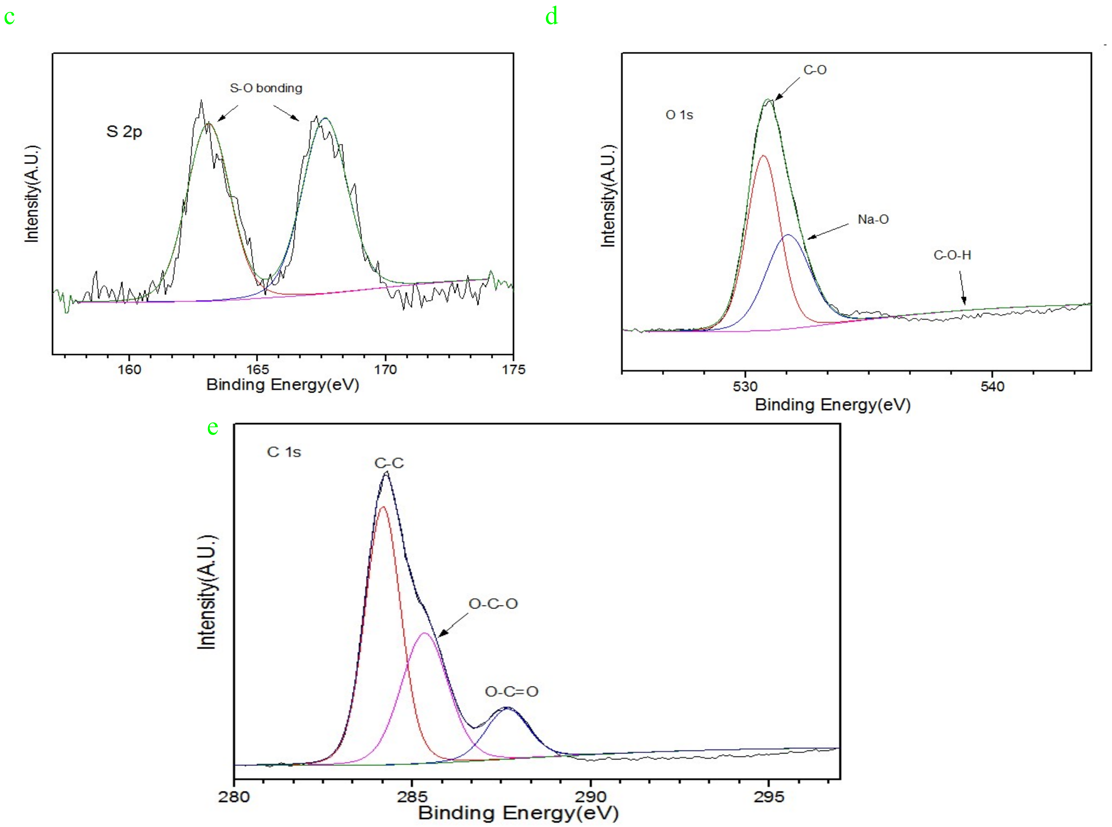

Figure S 13. XPS spectra of Sm-Na compound: (a) Na 1s; (b) Sm 3d; (c) S 2p ; (d) O 1s; (e) C 1s.

Figure S 12 shows the XPS spectra of Sm-Mn compound. Mn 2p XPS spectra show peaks at 641.1 eV, 645.8 eV and 653.3 eV. They are all related with Mn-O bonding.[s16-s18] They are assigned to Mn $2p_{3/2}$, Mn 2p $_{3/2}$ and Mn $2p_{1/2}$, respectively [s16-s18] Sm 3d XPS present two peaks at 1082.9 eV, 1109.9 eV, which is corresponding to Sm $3d_{5/2}$, Sm $3d_{3/2}$.[s2,s3] S 2p XPS spectra present peaks at 162.4 eV, 166.3 eV, which are assigned to S-O bonding.[s4,s5] O 1s XPS spectra show peaks at 531.2 eV, 532.3 eV, 537.2 eV, which are assigned to C-O, C-O-H, C-O-H bonding.[s10-s12] C 1s XPS spectra show peaks at 284.4 eV, 285.7 eV, 288.2 eV, which are considered to be C-C, O-C-O, O-C=O bonding. [s10]

Figure S13 shows XPS spectra of Sm-Na compound. Na 1s XPS spectra show a peak at 1070.2 eV, which is corresponding to Na-O bonding. [s19] Sm 3d XPS spectra show peaks at 1082.5 eV, 1109.2 eV, which are assigned to Sm $3d_{5/2}$, Sm $3d_{3/2}$.[s2,s3] S 2p XPS spectra show peaks at 163.1 eV, 167.7eV, which are due to S-O bonding.[s4,s5] O 1s XPS spectra present peaks at 530.9 eV, 531.7 eV and 539.9 eV, which are related with C-O, Na-O and C-O-H bonding.[s10-s12] C 1s

XPS spectra reveal peaks at 284.3 eV, 285.4 eV and 287.7 eV, which are assigned to C-C, C-O-C, C-O=C bonding.[s10]

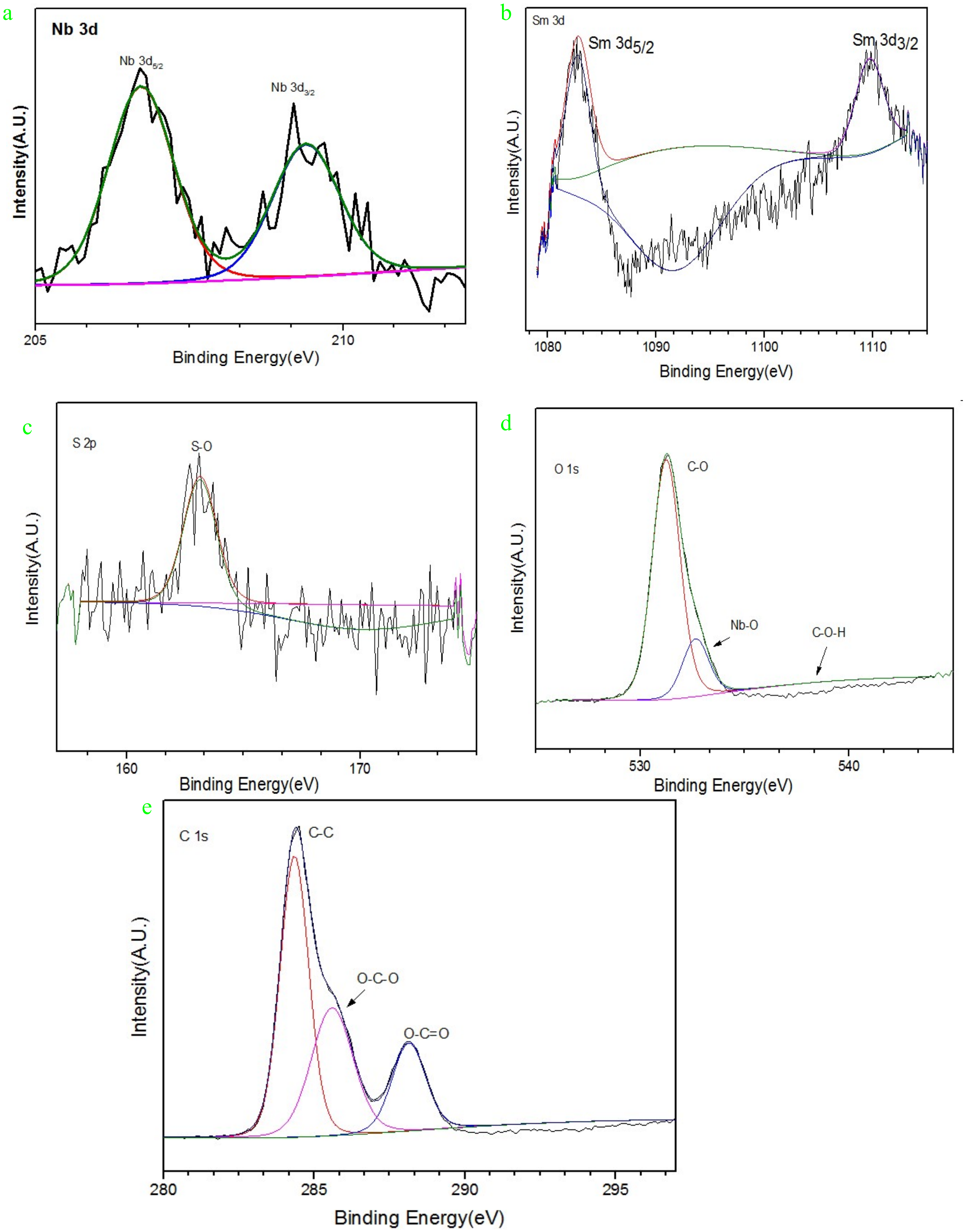

Figure S 14. XPS spectra of Sm-Nb compound : (a) Nb 3d; (b) Sm 3d; (c) S 2p; (d) O 1s; (e) C 1s.

Figure S 14 shows XPS spectra of Sm-Nb compound. Nb 3d XPS spectra show peaks at 206.7 eV and 209.4 eV, which are assigned to Nb $3d_{5/2}$ [s20] and Nb $3d_{3/2}$.[s21] Sm 3d XPS spectra present peaks at 1082.8 eV and 1109.7 eV, which are corresponding to Sm $3d_{5/2}$ and Sm $3d_{3/2}$.[s2,s3] S 2p XPS show a peak at 163.1 eV, which is assigned to S-O bonding. [s4,s5] O 1s XPS

spectra present peaks at 531.2 eV, 532.7 eV and 539.7 eV, which are regarded as C-O, Nb-O, C-O-H bonding.[s10-s12] C 1s XPS spectra show peaks at 284.4 eV, 285.6 eV and 288.1 eV, which are assigned to C-C, O-C-O, O-C=O bonding.[s10]

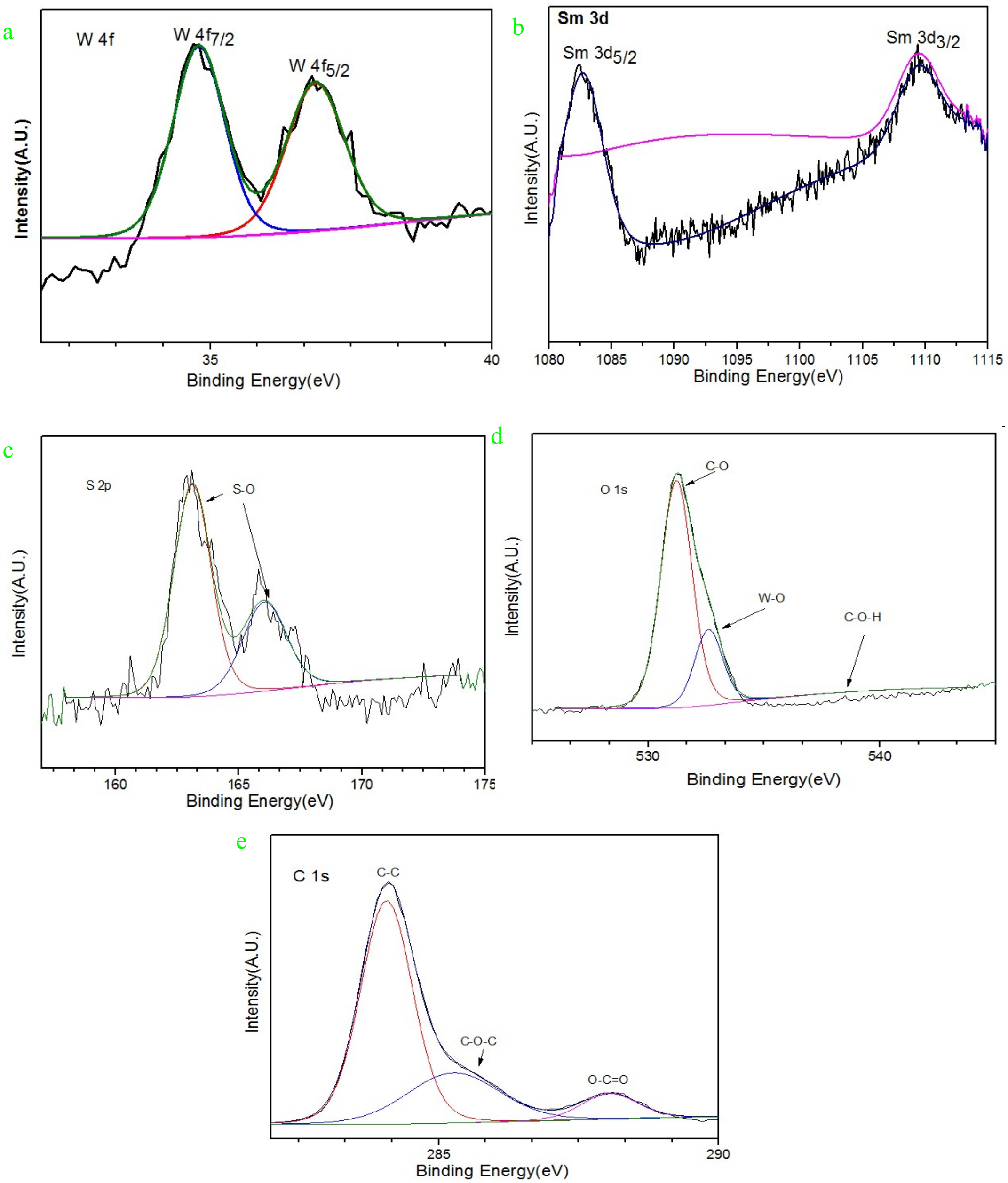

Figure S 15. XPS spectra of Sm-W compound: (a) W 4f; (b) Sm 3d; (c) S 2p ; (d) O 1s; (e) C 1s.

Figure S15 presents XPS spectra of Sm-W compound. W 4f XPS spectra show peaks at 34.8 eV and 36.9 eV. The peak at 34.8 eV is assigned to W $4f_{7/2}$ with W^{5+} state.[s22] The peak at 36.9 eV is assigned to W $4f_{5/2}$ with W^{5+} state. [s23] Sm 3d XPS spectra reveal peaks at 1082.6 eV and 1109.5 eV, which are considered as Sm $3d_{5/2}$ and Sm $3d_{3/2}$.[s2,s3] S 2p XPS spectra show peaks at 163.1 eV, 166.1 eV. Both are assigned to S-O bonding.[s4,s5] O 1s XPS spectra present peaks at 531.2 eV, 532.6 eV and 539.7 eV, which are considered as C-O, W-O, C-O-H bonding.[s10-s12] C 1s XPS spectra show peaks at, which are associated with C-C, O-C-O and O-C=O bonding.[s10]

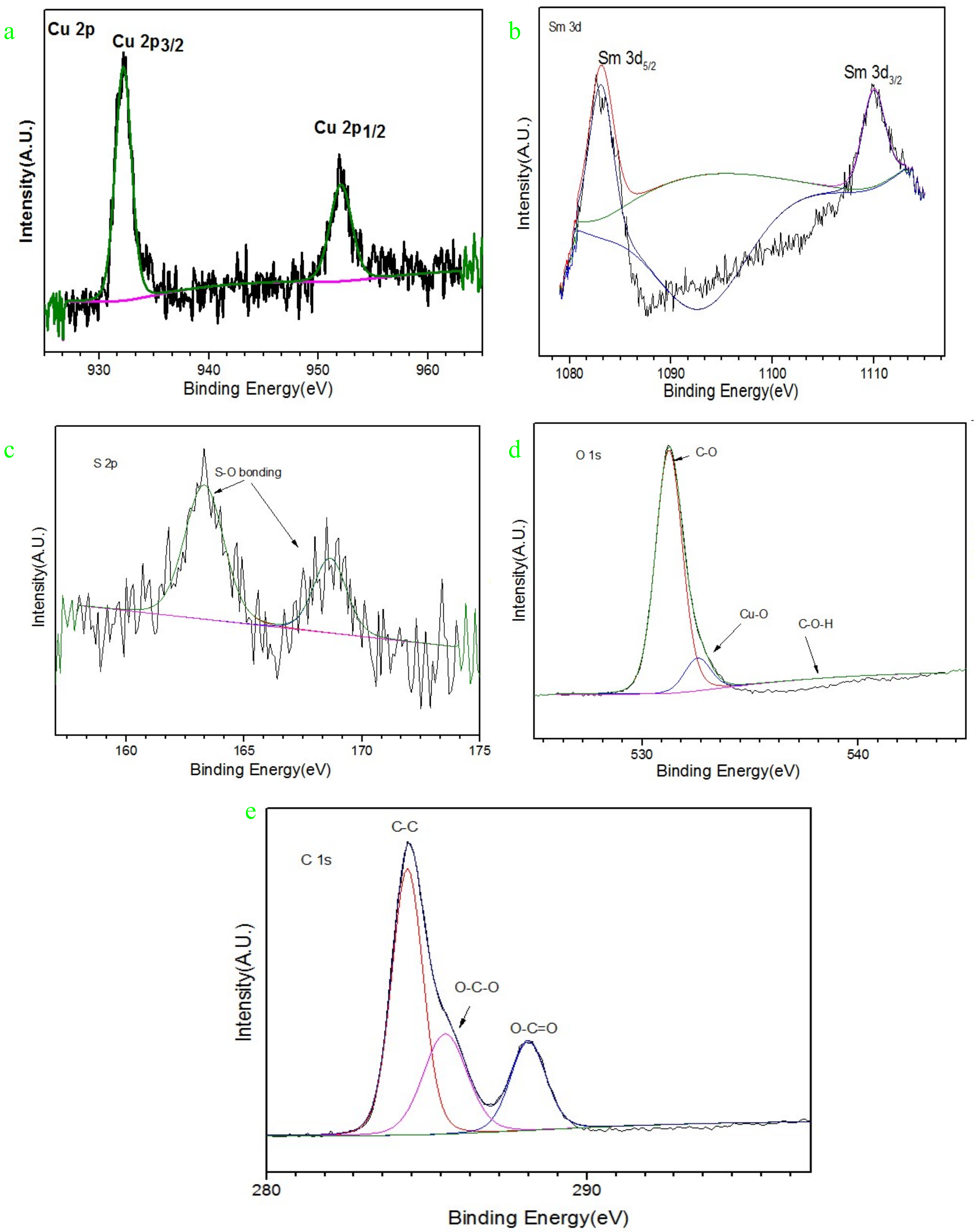

Figure S 16. XPS spectra of Sm-Cu compound: (a) Cu 2p ; (b) Sm 3d; (c) S 2p; (d) O 1s; (e) C 1s.

Figure S16 shows XPS spectra of Sm-Cu compound. Cu 2p XPS spectra show peaks at 932.2 eV, 952.2 eV, which are associated with Cu $2p_{3/2}$, Cu $2p_{1/2}$.[s24] Sm 3d XPS spectra present peaks at 1083.1 eV, 1110 eV, which are associated with Sm $3d_{5/2}$ and Sm $3d_{3/2}$.[s2,s3] S 2p XPS analysis show peaks at 163.4 eV and 168.7 eV , which are all assigned to S-O bonding.[s4,s5] O 1s XPS spectra present peaks at 531.3 eV, 532.6 eV and 538.9 eV, which are considered to be C-O, Cu-O and C-H-O bonding.[s10-s12] C 1s XPS spectra show peaks at 284.4 eV, 285.6 eV and 288.2 eV, which are associated with C-C, O-C-O, O-C=O bonding.[s10]

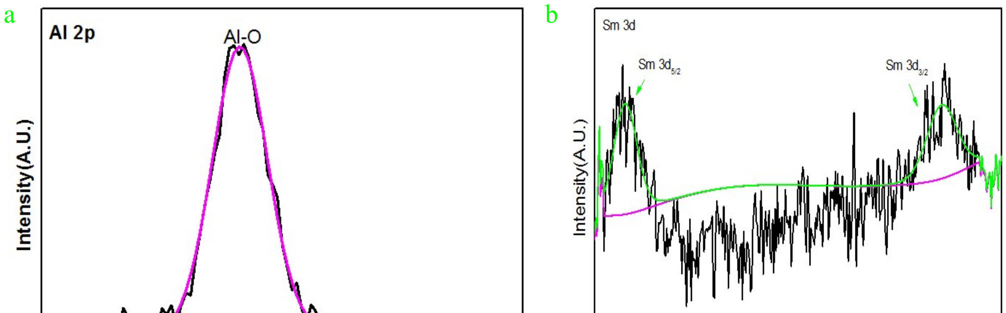

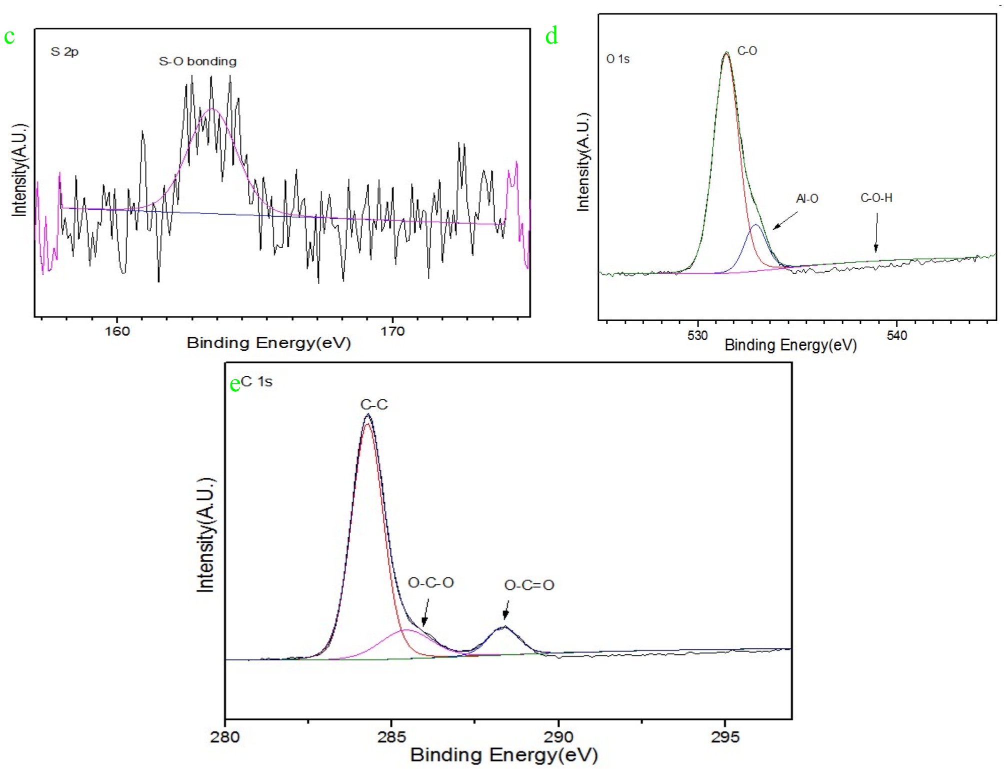

Figure S17. XPS spectra of Sm-Al compound: (a) Al 2p; (b) Sm 3d; (c) S 2p; (d) O 1s; (e) C 1s.

Figure S17 presents XPS spectra of Sm-Al compound. Al 2p XPS spectra show peak at 74.2 eV, which is related with Al-O bonding.[s25] Sm 3d XPS spectra reveal peaks at 1080.6 eV, 1109.8 eV, which are associated with Sm $3d_{5/2}$ and Sm $3d_{3/2}$.[s2,s3] S 2p XPS analysis show peaks at 163.4 eV, which is assigned to S-O bonding.[s4,s5] O 1s XPS spectra present peaks at 531.4 eV, 532.8 eV, and 539.1 eV, which are considered to be C-O, Al-O and C-H-O bonding.[s10-s12] C 1s XPS spectra show peaks at 284.3 eV, 285.5 eV and 288.4 eV, which are associated with C-C, O-C-O and O-C=O bonding.[s10]

Reference:

(s1) Yamashita, T.; Hayes, P.; P. Analysis of XPS spectra of Fe^{2+} and Fe^{3+} ions in oxide materials. *Appl. Surf. Sci.* 2008, 254, 2441-2449.

(s2) Suga, S.; Imada, S.; Ochiai, A.; Suzuki, T. XPS and BIS studies of electronic structures of Sm3Se 4 and Sm_4As_3. *Physica B* 1993, 186, 59-62.

(s3) Hillebrecht, F. U.; Fuggle, J. C. Invalidity of 4f count determination and possibilities for

determination of 4f hybridization in intermetallics of the light rare earths by core-level spectroscopy. *Phys. Rev. B* 1982, 25, 3550-3556.
(s4) Kelemen, S. R.; George, G. N.; Gorbaty, M. L. Direct determination and quantification of sulphur forms in heavy petroleum and coals 1. The X-ray photoelectron spectroscopy (XPS) approach. *Fuel* 1990, 69, 939-944.
(s5) Smart, R. St. C.; Skinner, W. M.; Gerson, A. R. XPS of Sulphide Mineral Surfaces: Metal-deficient, Polysulphides, Defects and Elemental Sulphur. *Surf. Interf. Anal*. 1999, 28, 101-105.
(s6) Tabet, N.; Faiz, M.; Al-Oteibi, A. XPS study of nitrogen-implanted ZnO thin films obtained by DC-Magnetron reactive plasma. *J. Electro. Spectro. Relat. Phenom*., 2008 163, 15-18.
(s7) Wei, D.; Liu, Y.; Wang, Y.; Zhang, H.; Huang, L.; Yu, G. Synthesis of N-Doped Graphene by Chemical Vapor Deposition and Its Electrical Properties. *Nano Lett.* 2009, 9, 5.
(s8) Li, X.; Wang, H.; Robinson, J. T.; Sanchez, H.; Diankov, G.; Dai; H. Simultaneous Nitrogen Doping and Reduction of Graphene Oxide. *J. Am. Chem. Soc.*, 2009, *131*, 15939-15944.
(s9) Zhang, L. -S.; Liang, X. -Q.; Song, W. -G.; Wu, Z. -Y. Identification of the nitrogen species on N-doped graphene layers and Pt/NG composite catalyst for direct methanol fuel cell. *Phys. Chem. Chem. Phys.* 2010, 12, 12055-12059.
(s10) Ganguly, A.; Sharma, S.; Papakonstantinou, P.; Hamilton, J. Probing the Thermal Deoxygenation of Graphene Oxide Using High-Resolution In Situ X-ray-Based Spectroscopies. *J. Phys. Chem. C* 2011, 115, 17009-17019.
(s11) Desimoni, E.; Casella, G. I. Morone, A.; Salvi; A. M. XPS Determination of Oxygen-containing Functional Groups on Carbon-fibre Surfaces and the Cleaning of These Surfaces. *Surf. Inter. Anal.*, 1990, 15, 627434.
(s12) Park, S. -J.; Jang, Y. -S. Pore Structure and Surface Properties of Chemically Modified Activated Carbons for Adsorption Mechanism and Rate of Cr(VI). J. Colloid Inter. Sci., 2002, 249, 458-463.
(s13) Wolter, S. D.; Luther, B. P.; Waltemyer, D. L.; Onneby, C.; Mohney, S. E. X-ray photoelectron spectroscopy and x-ray diffraction study of the thermal oxide on gallium nitride. *Appl. Phys. Lett.* 1997, 70, 2156-2158.
(s14) Powell, C. J. Elemental binding energies for X-ray photoelectron spectroscopy. *Appl. Surf. Sci.* 1995, 89,141-149.
(s15) Riskin, M.; Basnar, B.; Katz, E.; Willner, I. Cyclic control of the surface properties of a monolayer-functionalized electrode by the electrochemical generation of Hg nanoclusters. *Chem. Eur. J.* 2006, 12, 8549-8557.
(s16) DI CASTRO, V.; POLZONETTI, G. XPS study of MnO Oxidation. *J. Elect. Spectro. Relat. Pheno.* 1989, 48, 117-123.
(s17) A. Al-Agel, F.; Al-Arfaj, E.; A. Al-Ghamdi, A.; Losovyj, Y.; M. Bronstein, L.; E. Mahmoud, W. A novel recipe to improve the magnetic properties of Mn doped CeO_2 as a room temperature ferromagnetic diluted metal oxide. *J. Magn. Magn Mat.* 2014, 360, 73-79.
(s18) Wu, Z.; Jin, R.; Liu, Y.; H. Wang, H. Ceria modified MnOx/TiO_2 as a superior catalyst for NO reduction with NH_3 at low-temperature. *Catal. Comm.* 2008, 9, 2217–2220.
(s19) Kim, K. -J.; Kreider, P. B.; Choi, C.; Chang, C.-H.; Ahnb, H.-G. Visible-light-sensitive Na-doped p-type flower-like ZnO photocatalysts synthesized via a continuous flow microreactor. *RSC Adv.* 2013, 3, 12702-12710.

(s20) Atuchin, V. V.; Kalabina, I. E.; Keslerb, V. G.; Pervukhina. N. V. Nb 3d and O 1s core levels and chemical bonding in niobates. *J. Electron Spectro. Relat. Pheno.* 2005, 142, 129-134.
(s21) Zhao, F.; Wang, B.; Tang, Y.; Ge, H.; Huang, Z.; Liu, H. K. Niobium doped anatase TiO2 as an effective anode material for sodium-ion batteries. *J. Mater. Chem. A*, 2015, 3, 22969-22974.
(s22) Shpak, A. P.; Korduban, A. M.; Medvedskij, M. M.; Kandyba, V. O. XPS studies of active elements surface of gas sensors based on WO_{3-x} nanoparticles. *J. of Electron Spectro. Relat. Pheno.* 2007, 156-158, 172-175.
(s23) Yang, X. -L.; Dai, W. -L.; Gao, R.; Fan, K. Characterization and catalytic behavior of highly active tungsten-doped SBA-15 catalyst in the synthesis of glutaraldehyde using an anhydrous approach. *J. Catal.* 2007, 249, 278-288.
(s24)Abdulla-Al-Mamun, M.; Kusumoto, Y.; Muruganandham, M. Simple new synthesis of copper nanoparticles in water/acetonitrile mixed solvent and their characterization. *Mat. Lett.* 2009,63, 2007-2009.
(s25) Abrahami, S. T.; Hauffman, T.; de Kok, J. M. M.; Mol, J. M. C.; Terryn, H. XPS Analysis of the Surface Chemistry and Interfacial Bonding of Barrier-Type Cr(VI)-Free Anodic Oxides. *J. Phys. Chem. C* 2015, 119, 19967-19975.
